# Comparison of High-Efficiency MgO/Na_2_CO_3_ and MgO/K_2_CO_3_ as Heterogeneous Solid Base Catalysts for Biodiesel Production from Soybean Oil

**DOI:** 10.3390/molecules30132876

**Published:** 2025-07-07

**Authors:** Xiangyang Li, Xunxiang Jia, Weiji Li, Shufan Jia, Siwei Zhang, Jiliang Song, Jiao Wang

**Affiliations:** 1 Leicester International Institute, Dalian University of Technology, Panjin 124221, China; 1683367710@mail.dlut.edu.cn (X.J.); 1601084802@mail.dlut.edu.cn (W.L.); jsf13623633102@mail.dult.edu.cn (S.J.); zsw0076@mail.dlut.edu.cn (S.Z.); 2 School of Chemical Engineering, Ocean and Life Sciences, Dalian University of Technology, Panjin 124221, China; 3 Panjin Institute of Industrial Technology, Dalian University of Technology, Panjin 124221, China; wangjiao@dlut.edu.cn

**Keywords:** biodiesel production, solid base catalyst, transesterification reaction, magnesium oxide composite catalyst, sodium carbonate modification, potassium carbonate modification, soybean oil conversion

## Abstract

As a renewable alternative to fossil fuels, the industrial production of biodiesel urgently requires the development of efficient and recyclable solid base catalysts. In this study, the physicochemical properties and catalytic performance differences between MgO/Na_2_CO_3_ and MgO/K_2_CO_3_ catalysts were systematically compared using soybean oil as the raw material. By regulating the calcination temperature (500–700 °C), alcohol-to-oil ratio (3:1–24:1), and metal carbonate loading (10–50%), combined with N_2_ adsorption–desorption, CO_2_-TPD, XRD, SEM-EDS, and cycling experiments, the regulatory mechanisms of the ionic radius differences between sodium and potassium on the catalyst structure and performance were revealed. The results showed that MgO/Na_2_CO_3_-600 °C achieved a FAME yield of 97.5% under optimal conditions, which was 1.7% higher than MgO/K_2_CO_3_-600 °C (95.8%); this was attributed to its higher specific surface area (148.6 m^2^/g vs. 126.3 m^2^/g), homogeneous mesoporous structure, and strong basic site density. In addition, the cycle stability of MgO/K_2_CO_3_ was significantly lower, retaining only 65.2% of the yield after five cycles, while that of MgO/Na_2_CO_3_ was 88.2%. This stability difference stems from the disparity in their solubility in the reaction system. K_2_CO_3_ has a higher solubility in methanol (3.25 g/100 g at 60 °C compared to 1.15 g/100 g for Na_2_CO_3_), which is also reflected in the ion leaching rate (27.7% for K^+^ versus 18.9% for Na^+^). This study confirms that Na^+^ incorporation into the MgO lattice can optimize the distribution of active sites. Although K^+^ surface enrichment can enhance structural stability, the higher leaching rate leads to a rapid decline in catalyst activity, providing a theoretical basis for balancing catalyst activity and durability in sustainable biodiesel production.

## 1. Introduction

In the process of biodiesel industrialization, the reliance on homogeneous alkaline catalysts has led to significant cost increases and environmental challenges, primarily due to the generation of saponification byproducts during transesterification. These byproducts encapsulate active sites, necessitating multi-stage water washing processes that substantially increase wastewater treatment burdens and operational costs [1,2]. These technical challenges have driven researchers to turn their attention to solid alkali catalyst systems that can be separated and regenerated, among which magnesium oxide-based composites have attracted much attention due to their tunable surface alkalinity and thermal stability.

Recent studies have shown that the modification of magnesium oxide by alkali metal carbonates can effectively enhance the catalyst’s resistance to the negative impacts of free fatty acids [3,4]. Sodium and potassium ions, as commonly used cations for carbonate modification, have different atomic radii that may affect the formation of the pore structure and the distribution of basic sites on the catalysts, which in turn can lead to differences in catalytic performance. The utilization of waste or non-edible oils as feedstocks introduces additional challenges, primarily high free fatty acid content and contaminants such as moisture and metals, which can severely impact the effectiveness of base catalysts, whether homogeneous or heterogeneous, often necessitating costly pretreatment. Notably, the utilization of waste or non-edible oils (e.g., high-acid-value soybean oil [5,6,7]) introduces additional challenges, primarily high free fatty acid content and metal contaminants (e.g., Fe^2+^/Cu^2+^ [8,9]), which severely degrade base catalyst performance and necessitate costly pretreatment. Conversely, acidic catalysts or multifunctional catalysts containing acid sites—such as sodium montmorillonite [10], Mo/ZIF-8 [11], and Mo(VI)-based complexes [12]—leverage surface Brønsted and Lewis acid sites to catalyze both triglyceride transesterification and free fatty acid esterification in a single step, eliminating separate pretreatment.

However, the existing literature is mostly limited to the study of single modification systems and lacks a systematic analysis of the sodium/potassium ion synergistic mechanism and its dynamic behavior in soybean oil conversion. For instance, Shao et al. [13,14] developed an atomically dispersed magnesium catalyst with a three-fold increase in transesterification activity compared to the conventional catalysts, but did not address the multi-ion synergistic effect, while Krótki et al. [15] reported the zinc molybdate catalyst, which was capable of catalyzing simultaneous esterification/transesterification reactions, but were still limited to a single-active-site design.

This study investigates and compares the effects of sodium and potassium ion modification on the surface properties and catalytic stability of MgO/Na_2_CO_3_ and MgO/K_2_CO_3_ catalysts prepared via the impregnation–calcination method. This study reveals, for the first time, the regulatory mechanism of sodium/potassium ion size on the catalyst’s anti-carbon accumulation ability, which provides a theoretical basis for the development of multi-ion synergistic modification strategies.

## 2. Results and Discussion

The constitutive relationship between the physicochemical properties of solid alkali catalysts and their catalytic performance is the key to understanding the reaction mechanism. By systematically characterizing the surface properties, pore structure, and alkaline site distribution of different carbonate-modified magnesium oxide catalysts, the differentiated regulatory mechanisms of sodium and potassium ions on the carrier-active component interactions can be clarified to provide theoretical references for the development of high-performance biodiesel catalysts in order to promote the industrialization of green energy technology.

### 2.1. Phase and Surface Characterization

**Figure 1:**
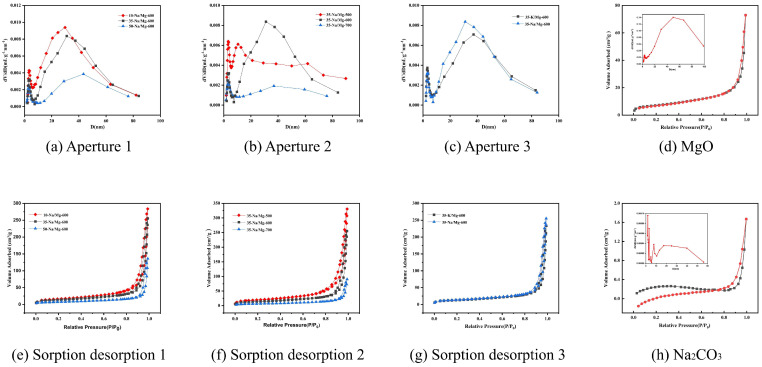
N_2_ adsorption–desorption isotherms (**e**–**h**) and pore size distribution (**a**–**d**): (**a**,**e**) 35%-Na_2_CO_3_/MgO-500 °C; (**b**,**f**) 35%-Na_2_CO_3_/MgO-600 °C; (**c**,**g**) 35%-K_2_CO_3_/MgO-600 °C; (**d**) MgO-600 °C; (**h**) Na_2_CO_3_.

To resolve the differences in physicochemical properties between MgO/Na_2_CO_3_ and MgO/K_2_CO_3_, this study combined various characterization tools to systematically analyze the surface structure, pore properties, and alkaline site distribution. The N_2_ adsorption–desorption isotherms and pore size distributions of MgO-600 °C and the carbonate-modified catalysts are shown in Figure 1.

In Figure 1, the N_2_ adsorption–desorption isotherm shows that 35%-MgO/Na_2_CO_3_-600 °C exhibits typical type IV mesoporous characteristics, with a significantly higher specific surface area (148.6 m^2^/g) and pore volume (0.42 cm^3^/g) than that of MgO/K_2_CO_3_ (126.3 m^2^/g, 0.35 cm^3^/g). The smaller ionic radius of Na^+^ (0.95 Å) makes it easier to embed in the MgO lattice interstitials to form a homogeneous mesoporous structure, whereas K^+^ (1.33 Å) is locally enriched on the surface due to spatial site resistance, leading to pore blockage (Table 1). The plain MgO-600 °C (without carbonate modification) shows a surface area of 105.4 m^2^/g, total pore volume of 0.51 cm^3^/g, and an average pore diameter of 9.8 nm, which can serve as a control for comparison. An analysis of the effect of calcination temperature further indicated that 600 °C was the optimal condition, when the MgO grain size was moderate and the pores were fully open.

**Figure 2:**
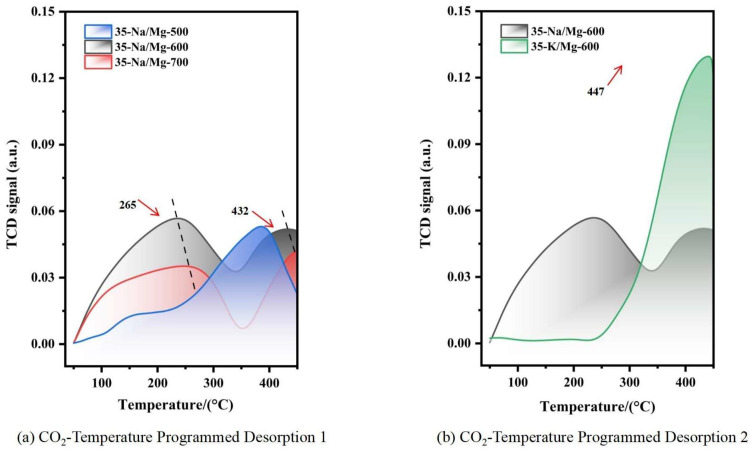
(**a**) CO_2_ desorption curves of 35%-Na_2_CO_3_/MgO at different calcination temperatures. (**b**) Difference in desorption peaks between Na_2_CO_3_- and K_2_CO_3_-supported catalysts calcined at 600 °C.

The comparison of CO_2_-TPD curves for different catalysts is shown in Figure 2.

In Figure 2, the CO_2_-TPD results demonstrate that MgO/Na_2_CO_3_ exhibits a significantly higher density of alkaline sites compared to MgO/K_2_CO_3_. The strong alkaline sites of MgO/Na_2_CO_3_, evidenced by distinct desorption peaks at 265 °C and 432 °C, suggest its prominent ability to form methoxide, which is responsible for the nucleophilic attack to the triglycerides. In contrast, the peak of MgO/K_2_CO_3_ appears at the position of 447 °C. Therefore, MgO/K_2_CO_3_ displayed inferior stability in cycling experiments and lower basicity.

Quality Control: XRD validation (JCPDS 45-0946, ±5% peak intensity tolerance) and BET analysis (±3 m^2^/g surface area fluctuation) ensured batch-to-batch consistency. Statistical rigor was maintained through triplicate experiments and Grubbs’ outlier rejection (95% confidence).

**Figure 3:**
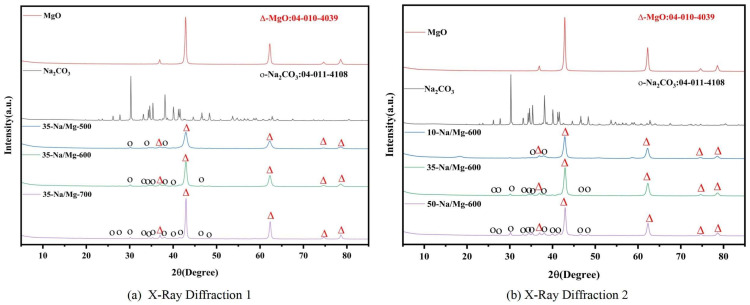
(**a**) XRD patterns of 35%-Na_2_CO_3_/MgO at different calcination temperatures. (**b**) XRD peak position differences of Na_2_CO_3_ catalysts calcined at 600 °C.

The comparison of XRD patterns of different catalysts is shown in Figure 3.

**Figure 4:**
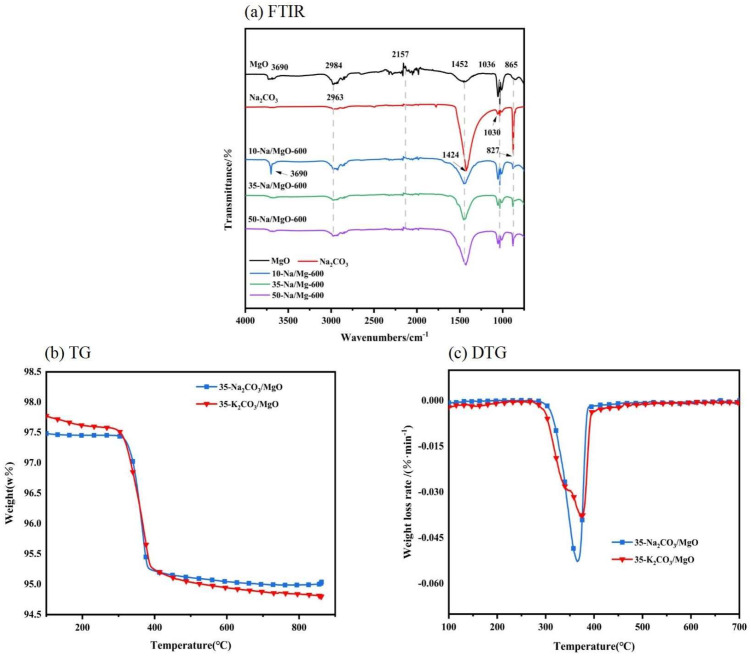
(**a**) FTIR spectra. (**b**) TG-DTG weight curve of Na_2_CO_3_/K_2_CO_3_. (**c**) TG-DTG weight loss curve of Na_2_CO_3_/K_2_CO_3_.

In Figure 3, the XRD pattern further verifies the crystal structure difference in the two catalysts. The weak diffraction peaks (corresponding to the crystal plane of Na_2_CO_3_) appear at 2θ = 32.1°and 46.5°, indicating that the active components are highly dispersed in the form of microcrystals; however, the intensity of the K_2_CO_3_ diffraction peak of MgO/K_2_CO_3_ is higher, indicating that it has higher crystallinity and poor dispersion. In addition, the insertion of Na^+^ into the MgO lattice resulted in the shift in the (111) crystal plane peak position (36.9° → 37.0°) and the reduction in the lattice constant (4.209 Å), which enhanced the binding force between the carrier and the active component and inhibited the dissolution of Na. In contrast, K^+^ is distributed on the surface because it cannot be embedded in the lattice, leading to weak interfacial bonding, which can easily cause the loss of active sites in the reaction due to scouring. The combination diagram of FTIR and TG-DTG for the catalyst is shown in Figure 4.

In Figure 4, FTIR and TG-DTG analyses reveal the differences in the surface chemical properties and thermal stability of the catalysts. The strong hydroxyl peak at 3690 cm^−1^ for MgO/Na_2_CO_3_ indicates that the surface is rich in free hydroxyl groups, which might promote reactant adsorption, while the hydroxyl peak of MgO/K_2_CO_3_ shows a 32% decrease in intensity, suggesting the shielding of surface hydrophilicity by K^+^. The TG-DTG curves show that the total weight loss of MgO/K_2_CO_3_ (15.0%) is lower than that of MgO/Na_2_CO_3_ (17.9%), and the surface hydroxides and carbonates decomposition temperature is higher (410 °C vs. 385 °C), confirming that its thermal stability is superior. This property resulted in less structural damage during regeneration at high temperatures, thus maintaining a higher activity retention rate in recycling.

In conclusion, the high specific surface area (148.6 m^2^/g), homogeneous mesoporous structure, and abundant basic sites of MgO/Na_2_CO_3_ contribute to its superior initial FAME yield (97.5%) in the transesterification reaction.

### 2.2. Catalyst Characterization and Performance Testing

(1) Characterization Method Based on Energy Spectrum Analysis (SEM-EDS)

To analyze the composition and distribution of elements on the catalyst surface, experiments were carried out using a scanning electron microscope (SEM) equipped with an energy spectrometer (EDS) coupling technique (model: Hitachi SU8010, Hitachi, Japan) for characterization. Specific steps included sample preparation, testing conditions, and data analysis.

Sample preparation: The dried catalyst powder was uniformly dispersed on conductive adhesive tape, and a 5 nm gold film (thickness calibrated by a quartz crystal microbalance) was obtained by using an ion sputtering apparatus (E-1045, Hitachi, Ltd.) sprayed at a current of 15 mA for 60 s to improve the electrical conductivity and to reduce the charge accumulation.

Testing conditions: The SEM accelerating voltage was set to 15 kV, the working distance was 10 mm, and the sample morphology was observed in secondary electron (SE) mode. Multiple representative areas were selected for spot scanning or surface scanning during EDS inspection, and the inspection time for each area was 60 s to ensure the stability of the X-ray signal.

**Figure 5:**
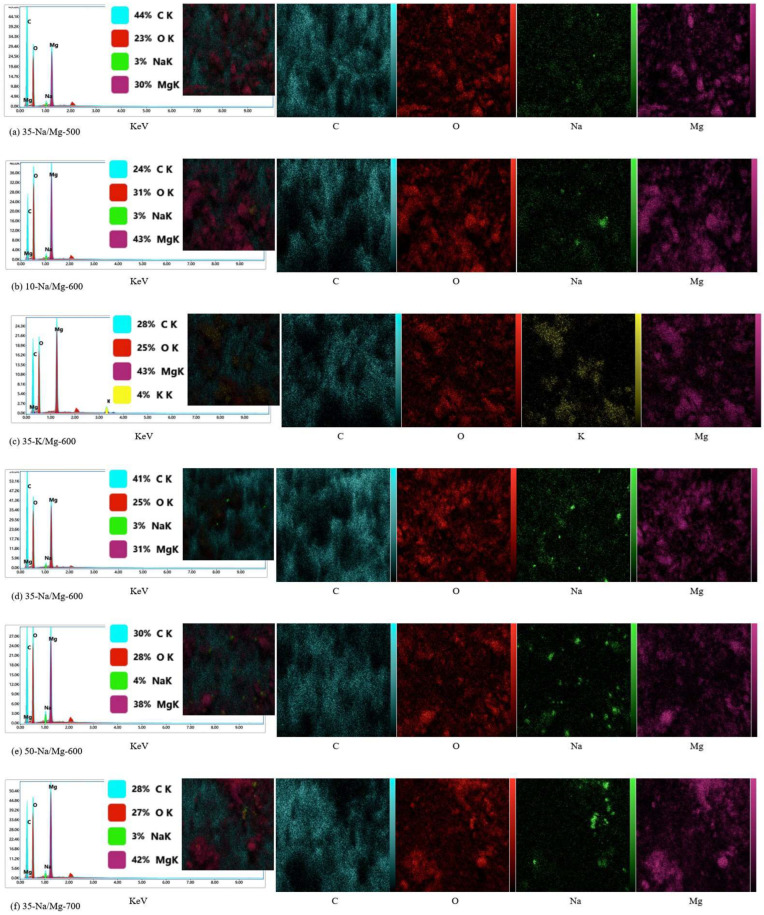
EDS mapping-related images.

Data analysis: The collected X-ray spectra were analyzed qualitatively and semi-quantitatively using the EDS software (Oxford Instruments AZtec), and the atomic percentage (At.%) and mass percentage (wt%) of each element were calculated. At the same time, elemental distribution maps were obtained using the mapping function to assess the homogeneity of the active components such as Na, K, and Mg on the catalyst surface. The EDS mapping-related images are shown in Figure 5.

**Figure 6:**
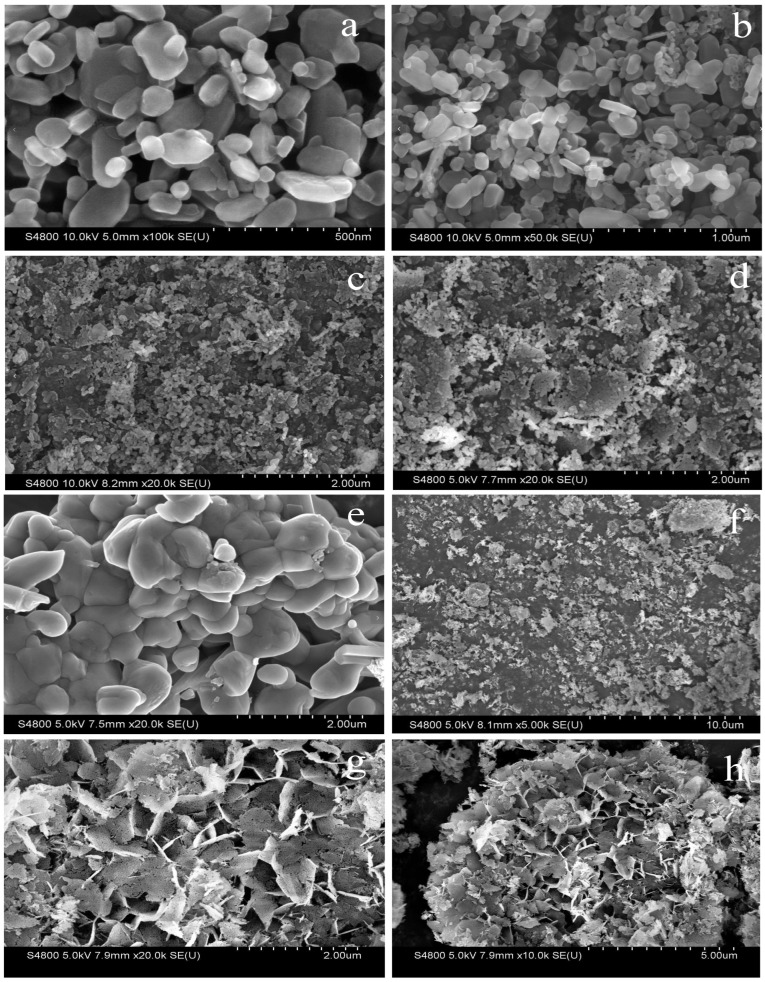
SEM images. Note: (**a**) 100k-35-Na/Mg-600; (**b**) 50k-35-Na/Mg-600; (**c**) 20k-35-Na/Mg-700; (**d**) 20K/MgO; (**e**) 20k-Na_2_CO_3_; (**f**) 5k-50-Na/Mg-600; (**g**) 20k-35-K/Mg-600; (**h**) 10k-35-K/Mg-600.

In Figure 5, the EDS mapping results and X-ray energy spectrum analysis of Na, K, Mg, and O elements on the catalyst surface are shown. The distribution of each element on the catalyst surface can be clearly observed on the mapping images. The distribution of Na in each region is more uniform, indicating its uniform existence on the whole catalyst surface. Its mass percentage (wt%) and atomic percentage (At.%) are 1.58% and 2.10%, respectively, according to the X-ray spectra. The distribution of K is similar to that of Na, being uniformly distributed without obvious aggregation. Its mass percentage and atomic percentage are 2.32% and 1.98%, respectively. The distribution of Mg is slightly uneven, but still relatively homogeneous on the whole, with stronger signals in individual regions, which may experience a local enrichment phenomenon. Its mass percentage and atomic percentage are 0.88% and 0.75%, respectively. O is the most evenly distributed element, covering the whole catalyst surface, indicating the presence of more oxides on the catalyst surface. Its mass percentage and atomic percentage are 54.12% and 63.56%, respectively. The above data were quantitatively analyzed by means of Oxford Instruments AZtec software, which showed that the elements Na, K, and Mg are more uniformly distributed on the catalyst surface, and O covers the whole surface, providing an idea of the composition and uniformity of the distribution of the elements on the catalyst surface. Example SEM images are shown in Figure 6.

In Figure 6, SEM images of different catalysts at different magnifications are shown. Figure 6a,b show 100 k× and 50 k× magnification images of the 35-Na/Mg-600 sample, respectively. Figure 6b shows that the catalyst particles are regular granular particles with a smooth surface and large specific surface area. In Figure 6a, at a higher magnification, the details of the particles are more obvious and the interstices become larger, indicating that the particles have agglomerated to some extent. Figure 6c,d show 20 k× magnifications of the 35-Na/Mg-700 and pure MgO. It is evident that at the high-temperature calcination condition of 700 °C, agglomeration occurs on the particle surface, which may lead to a reduction in the specific surface area of the catalyst and consequently decrease its catalytic activity. Figure 6e shows an image of pure Na_2_CO_3_ at 20 k×, demonstrating a lamellar structure. Figure 6f shows the 50-Na/Mg-600 sample at 5000× magnification. The 35-K/Mg-600 sample (Figure 6g, 20 k×) exhibits larger particle aggregates (2–5 μm vs. 0.5–2 μm for Na) with pronounced surface fractures, attributed to K_2_CO_3_’s lower melting point (891 °C) inducing partial fusion during calcination. At 10 k× magnification (Figure 6h), these aggregates show wider interparticle gaps and dendritic pore channels, suggesting that potassium modification enhances macroporosity but reduces mechanical stability.

(2) Scope of Performance Testing

Catalyst performance evaluation focused on 35 wt% loading at 600 °C (35-Na/Mg-600 and 35-K/Mg-600) to ensure parameter uniformity for direct Na^+^/K^+^ comparison. Potassium-based samples with other conditions (e.g., 35-K/Mg-500) were excluded from catalytic testing to reduce the complexity of the analysis. Table 2 presents the catalyst matrix used for systematic optimization, as detailed below.

### 2.3. Catalytic Performance Conditions

#### 2.3.1. Effect of Loading Rate for MgO/Na_2_CO_3_

Experimental conditions:

- Fixed parameters: Temperature = 60 °C; catalyst consumption = 4.0 wt%; methanol-to-oil ratio = 12:1; reaction time = 3 h.
- Variable parameter: Sodium carbonate content (10–50%).

**Figure 7:**
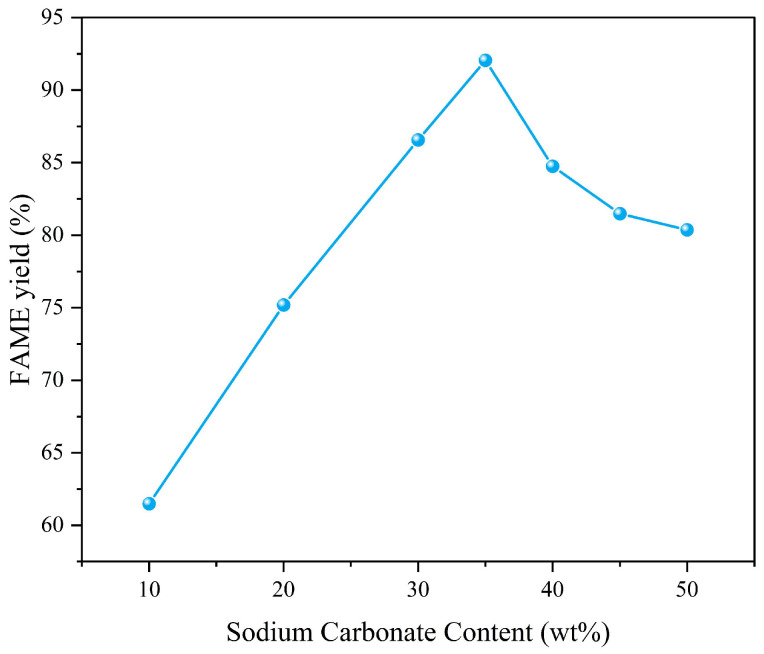
Relationship between loading rate for Na_2_CO_3_-MgO and FAME yield.

Analysis:

- Optimal range: Increasing the loading rate from 10% to 35% enhanced the FAME yield from 61.5% to 92.1% (Figure 7). This improvement can be attributed to the enhanced overall activity of the catalyst as the Na_2_CO_3_ content increases, further suggesting that MgO alone does not exhibit exceptionally strong catalytic properties.
- Decline at high sodium carbonate content: Further increasing the loading rate to 50% reduced the yield to 80.3%, which can be attributed to the excessively high loading rate that resulted in a reduction in the catalyst’s specific surface area.
- Considering that the increase in sodium carbonate content above 35% did not bring about an improvement in yield, we chose 35% as the optimal loading rate.
- The effect of the loading rate for Na_2_CO_3_-MgO on the FAME yield is shown in Figure 7.

#### 2.3.2. Effect of Calcination Temperature for MgO/Na_2_CO_3_

Experimental conditions:

- Fixed parameters: Temperature = 60 °C; catalyst consumption = 3.0 wt%; methanol-to-oil ratio = 12:1; reaction time = 2 h.
- Variable parameter: Calcination temperature for catalysts (500–700 °C).

**Figure 8:**
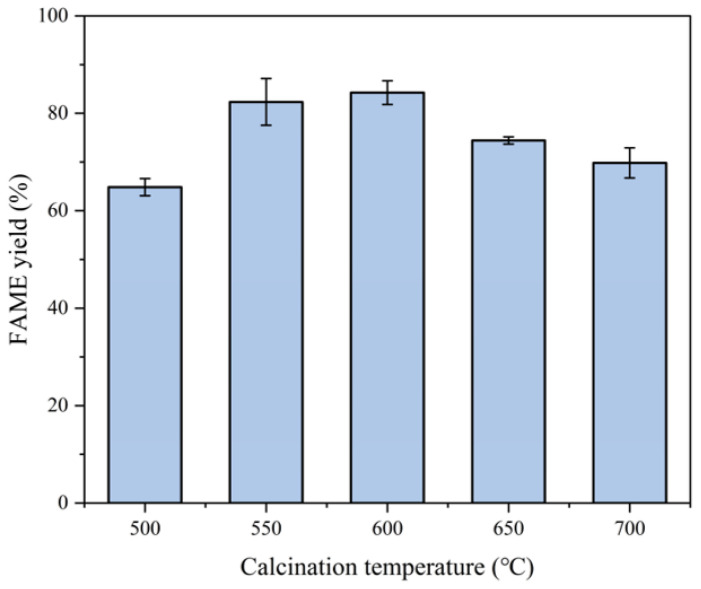
Relationship between calcination temperature for MgO/Na_2_CO_3_ and FAME yield (error bars: ±SD, *n* = 3).

Analysis:

- Optimal range: When the calcination temperature increased from 500 to 600 °C, the FAME yield significantly rose from 64.8% to 84.2%. According to the BET and CO_2_-TPD characterization results in Figure 1a and Figure 2b, MgO/Na_2_CO_3_ calcined at 600 °C exhibited the highest catalytic activity and a relatively large specific surface area, which contributed to the substantial increase in yield. Due to the decreased amount of the catalyst and the shortened reaction time, the overall yield in this group was relatively low. Therefore, the corresponding yield changes of different catalysts can be clearly distinguished.
- Decline at high calcination temperature: Further increasing the calcination temperature to 700 °C reduced the yield to 69.8%. It is evident from the SEM in Figure 6 and BET in Figure 1b that an excessively high calcination temperature causes the agglomeration of the catalyst structure, thereby reducing the specific surface area and ultimately leading to a decrease in the FAME yield.
- The slightly higher specific surface area observed for the 500 °C sample (112.4 m^2^/g) compared to the 700 °C sample (98.5 m^2^/g) can be attributed to incomplete sintering at lower temperatures, preserving a more open pore structure. However, optimal catalytic activity was achieved at 600 °C due to balanced MgO crystal facet activation and uniform dispersion of carbonate species, maximizing strong alkaline site density, as evidenced by CO_2_-TPD. Calcination at 700 °C induced severe sintering, collapsing mesopores, and reducing accessibility. This temperature-dependent structural evolution aligns with findings for other mineral-based catalysts, where 600 °C represents the optimal window for achieving high surface area, developed porosity, and sufficient base strength critical for transesterification [16,17,18,19,20,21].
- Based on the experimental data and characterization results, it is reasonable to conclude that 600 °C represents the optimal calcination temperature.
- The effect of calcination temperature for MgO/Na_2_CO_3_ on the FAME yield is shown in Figure 8.

#### 2.3.3. Effect of Reaction Temperature

Experimental conditions:

- Fixed parameters: Catalyst consumption = 4.0 wt%; methanol-to-oil ratio = 12:1; reaction time = 4 h.
- Variable parameter: Temperature (30–80 °C).

**Figure 9:**
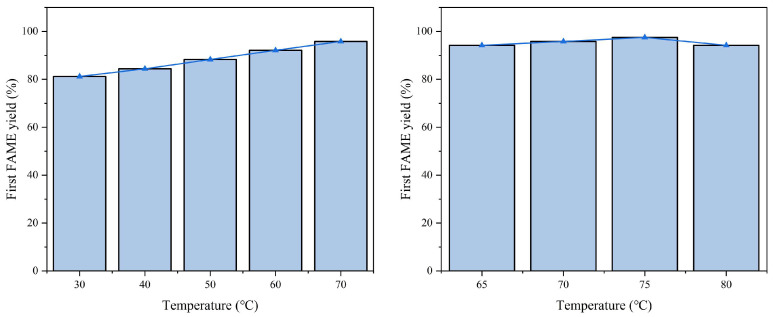
Relationship between reaction temperature and FAME yield (*n* = 3).

Analysis:

- Optimal range: Increasing the temperature from 30 °C to 70 °C enhanced the FAME yield from 81.2% to 95.8% (Figure 9). This improvement is attributed to the accelerated mass transfer and reaction kinetics at elevated temperatures.
- Decline at high temperatures: Further increasing the temperature to 80 °C reduced the yield to 94.2%, likely due to methanol volatilization and partial catalyst sintering.
- Considering that the increase in temperature above 65 °C did not bring about a significant improvement in yield in terms of environmental protection and productivity, we chose 65 °C as the subsequent reaction condition.

The effect of reaction temperature on the first-round reaction FAME yield is shown in Figure 9.

#### 2.3.4. Effect of Catalyst Consumption

Experimental Conditions:

- Fixed parameters: Temperature = 65 °C; methanol-to-oil ratio = 12:1; reaction time = 3 h.
- Variable parameter: Catalyst consumption (1.0–6.0 wt%).

**Figure 10:**
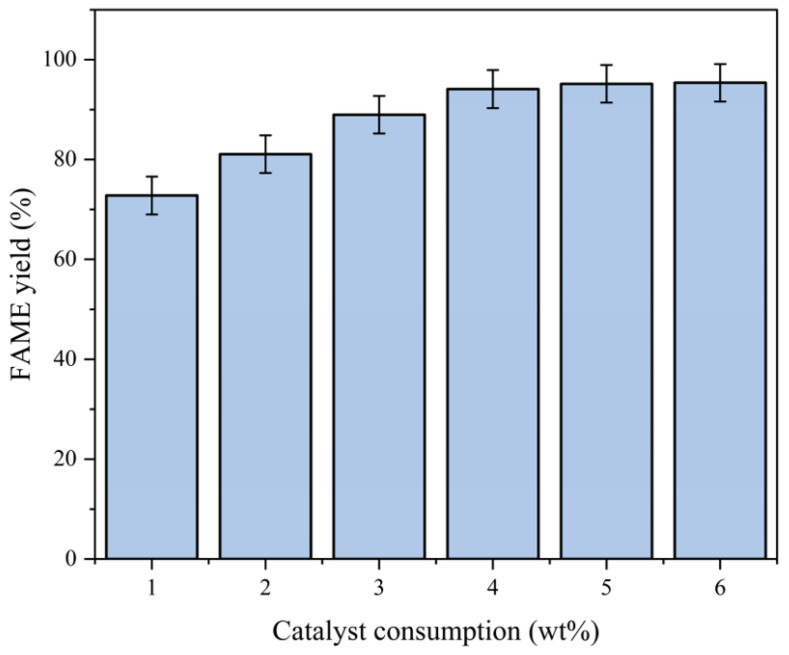
Relationship between catalyst dosage and FAME yield (error bar: ±SD, *n* = 3).

Analysis:

- Optimal loading: The FAME yield increased from 73.0% to 94.3% as the catalyst consumption rose from 1.0 wt% to 4.0 wt% (Figure 10).
- Overloading issues: Excessive loading (6.0 wt%) did not bring about a significant increase in yield, likely due to emulsion formation or active site shielding.
- Mechanism:
- Higher catalyst consumption provides more active sites for methanol activation.
- For the comprehensive consideration of economic efficiency and yield, we chose 4.0 wt% as the subsequent catalyst consumption.
- Overloading increases viscosity, hindering reactant diffusion to active sites.
- The effect of catalyst loading on FAME yield is shown in Figure 10.

#### 2.3.5. Effect of Methanol-to-Oil Ratio

Experimental conditions:

- Fixed parameters: Temperature = 65 °C; catalyst consumption = 4.0 wt%; reaction time = 2 h.
- Variable parameter: Methanol-to-oil ratio (3:1–24:1).

**Figure 11:**
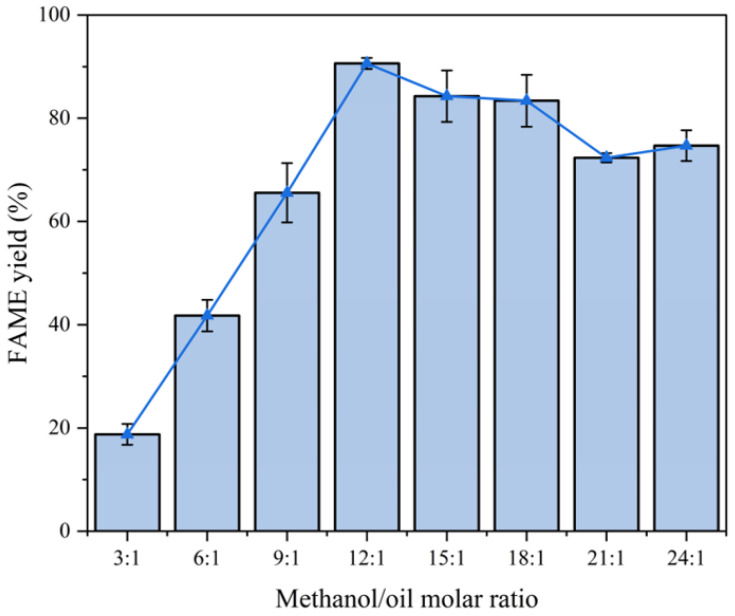
Methanol-to-oil ratio effects on FAME yield (error bars: ±SD, *n* = 3).

Analysis:

- Optimal ratio: Increasing the ratio from 3:1 to 12:1 improved the yield by 72.8% (18.8% to 91.6%) (Figure 11).
- Dilution effect: A higher ratio (15:1) reduced efficiency due to reactant dilution.
- Mechanism:
- Excess methanol shifts the equilibrium toward ester formation but dilutes triglycerides at extreme ratios.
- For the comprehensive consideration of economic efficiency and yield, we chose 12:1 as the subsequent methanol/oil molar ratio.
- The relationship between methanol/oil molar ratio and FAME yield is shown in Figure 11.

#### 2.3.6. Effect of Reaction Time

Experimental conditions:

- Fixed parameters: Temperature = 65 °C; catalyst consumption = 4.0 wt%; methanol-to-oil ratio = 12:1.
- Variable parameter: Reaction time (1–4 h).

**Figure 12:**
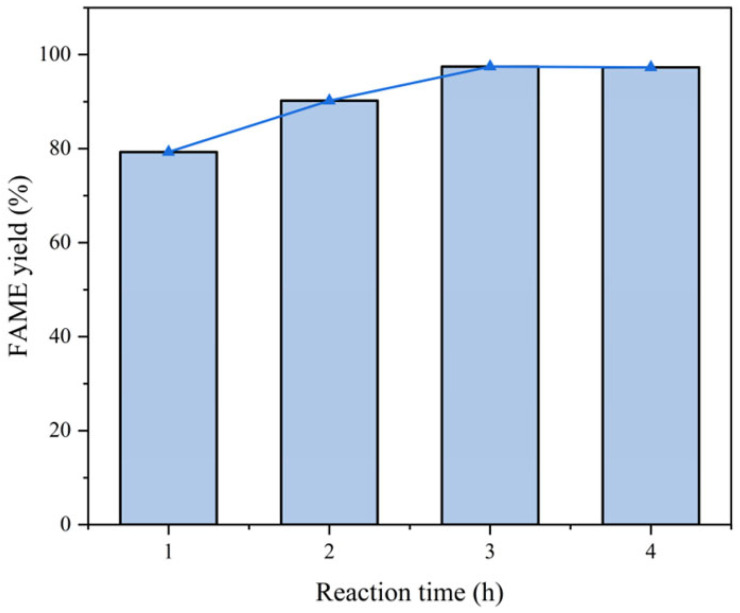
Reaction time effects on FAME yield.

Analysis:

- Optimal duration: Extending the reaction time from 1 h to 3 h increased the yield from 79.3% to 97.4% (Figure 12).
- Reverse reactions: Prolonged time (>3 h) intensified reverse esterification, leading to no significant increase in the yield.
- Mechanism:
- Longer time ensures complete conversion but risks glycerol adsorption on active sites. Considering energy conservation and economic benefits, we chose 3 h as the optimal reaction time.
- The relationship between reaction time and FAME yield is shown in Figure 12.

### 2.4. Circulation and Regeneration

**Figure 13:**
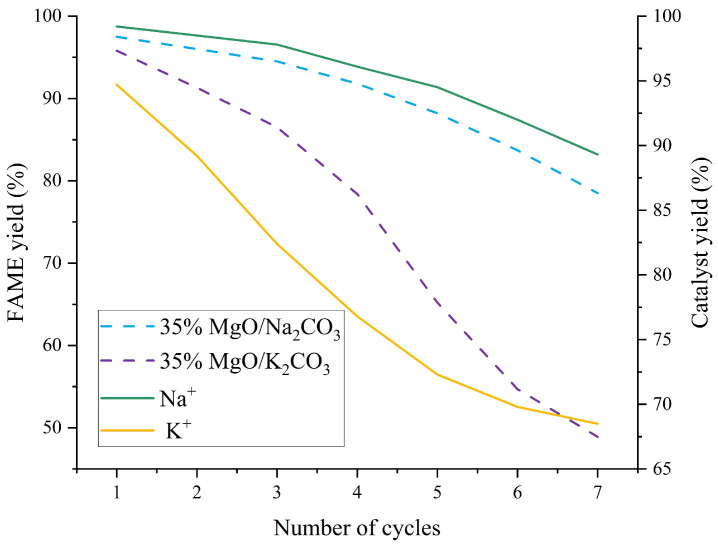
Cyclic stability of MgO/Na_2_CO_3_ and MgO/K_2_CO_3_ catalysts.

The cycling performance of MgO/Na_2_CO_3_ and MgO/K_2_CO_3_ catalysts was compared under optimized conditions (65 °C, 4.0 wt% catalyst, 12:1 methanol-to-oil ratio, 3 h). The cyclic stability of MgO/Na_2_CO_3_ and MgO/K_2_CO_3_ catalysts is shown in Figure 13.

Figure 13 compares the cyclic stability of MgO/Na_2_CO_3_ and MgO/K_2_CO_3_ catalysts under optimized conditions (65 °C, 4.0 wt% loading, 12:1 methanol-to-oil ratio, 3 h). MgO/Na_2_CO_3_ exhibits a high initial FAME yield of 97.5%, which gradually declines to 78.5% after seven cycles (activity loss of 19.0%). In contrast, MgO/K_2_CO_3_ shows a lower initial yield (95.8%) but a more rapid deactivation, reaching 48.9% after seven cycles (activity loss of 46.9%). This divergence stems from structural differences: Na_2_CO_3_-modified catalysts retain higher porosity and active site density, enabling gradual performance decay, while K_2_CO_3_’s dense crystalline phase initially resists leaching but suffers from irreversible structural collapse under prolonged reaction stress. The cumulative Na^+^ leaching rate (18.9% after five cycles) is lower than K^+^’s (27.7%), yet MgO/Na_2_CO_3_’s higher solubility in methanol (1.15 g/100 g) accelerates active component loss over time.

**Figure 14:**
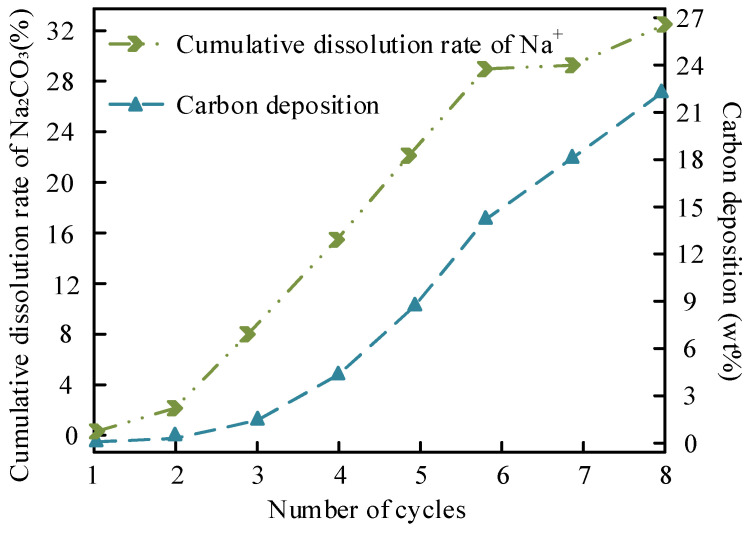
Na^+^ leaching and carbon deposition in MgO/Na_2_CO_3_.

The leaching of Na^+^ and carbon deposition in the MgO/Na_2_CO_3_ system are shown in Figure 14.

Figure 14 illustrates the relationship between Na^+^ leaching and carbon deposition in MgO/Na_2_CO_3_ during cycling. The Na^+^ leaching rate increases linearly with cycle number, reaching 18.9% after five cycles, while carbon deposition accumulates to 22.3 wt% by the seventh cycle. The relatively high solubility of Na_2_CO_3_ in methanol facilitates ion dissolution, leaving vacant sites prone to carbonaceous byproduct adsorption. FTIR analysis (Figure 4a) confirms that free hydroxyl groups on MgO/Na_2_CO_3_ (peak at 3690 cm^−1^) enhance reactant adsorption but also promote glycerol polymerization, contributing to pore blockage. Despite these challenges, the homogeneous mesoporous structure partially mitigates mass transfer limitations, allowing gradual rather than abrupt deactivation.

**Figure 15:**
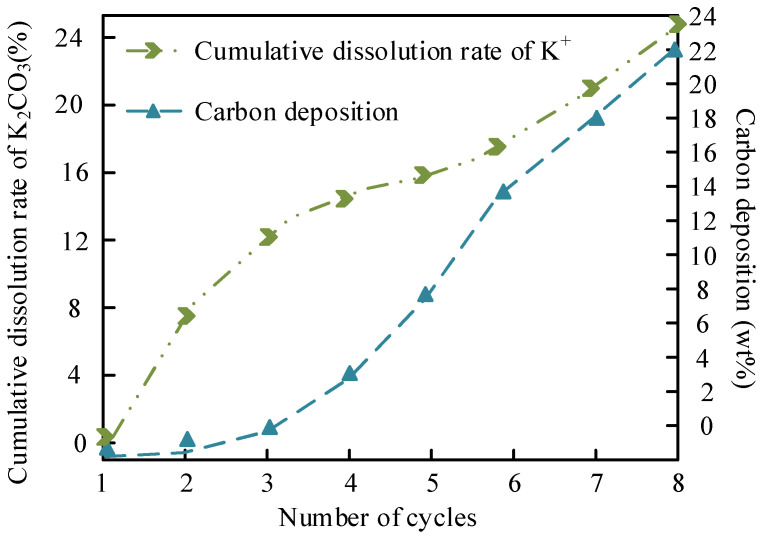
K+ leaching and carbon deposition in MgO/K_2_CO_3_.

The leaching of K+ and carbon deposition in MgO/K_2_CO_3_ are shown in Figure 15.

Figure 15 details the K^+^ leaching and carbon deposition behavior in MgO/K_2_CO_3_. Because of K_2_CO_3_’s higher solubility in methanol (3.25 g/100 g at 60 °C), the cumulative K^+^ leaching rate after five cycles is 27.7%, higher than that of Na^+^ (18.9%), due to the formation of a stable crystalline K_2_CO_3_ phase on MgO. However, carbon deposition (16.8 wt% after seven cycles) occurs more rapidly, driven by the hydrophobic surface of K_2_CO_3_ (reduced hydroxyl peak intensity in FTIR), which favors triglyceride adsorption but hinders glycerol removal. The dense structure of MgO/K_2_CO_3_ initially resists pore clogging but eventually leads to irreversible active site coverage, causing a steep decline in the FAME yield. This trade-off highlights the need for balancing solubility and structural robustness in catalyst design.

### 2.5. Comparative Literature

#### 2.5.1. Structural Design of MgO-Based Composite Catalysts

Recent advancements in solid base catalysts have focused on optimizing the interfacial engineering and structural stability of magnesium oxide (MgO)-based composites. Wang et al. [16] demonstrated that tailored ionic conduction pathways in boronic ester transesterification significantly enhance proton migration rates, establishing a theoretical foundation for high-activity catalyst design. Metal doping strategies, such as Al^3+^ substitution in Fe-based oxides, have been proven effective in modulating basic site distributions. Ikeue et al. [17] reported a 2.3-fold increase in surface basicity density via Al^3+^ substitution, offering critical insights for constructing MgO/carbonate hybrid systems. Bernard et al. [18] further elucidated the structural stabilization mechanism of MgO/Na_2_CO_3_ composites, where hydrotalcite phase formation (Mg_6_Al_2_CO_3_(OH)_16_·4H_2_O) under high-temperature conditions effectively mitigates sintering. Rostamizadeh et al. [11] highlighted the synergistic effects of porous carriers and active components, achieving a five-fold acceleration in esterification kinetics through ZIF-8-supported molybdenum catalysts. These studies collectively emphasize the importance of interfacial bonding optimization for catalytic performance enhancement.

#### 2.5.2. Catalytic Performance in Biodiesel Production

The catalytic efficiency and recyclability of solid base catalysts are critical for industrial biodiesel synthesis. Sodium-based montmorillonite catalysts, as reported by She et al. [10], exhibited exceptional stability, with only 0.8 wt% sodium loss after 10 cycles, attributed to strong interlayer polarization effects. In contrast, potassium-based systems face severe limitations due to K_2_CO_3_’s high solubility (>1.8 g/100 mL in methanol), which triggers ion dissolution and equipment fouling. Lobo et al. [19], through comparative experiments on magnesium metabolism in dairy feed, found that the smaller hydration radius of potassium ions (3.31 Å vs. 3.58 Å for sodium ions) resulted in easier de-embeddedness from the carrier skeleton, a biological finding which provides an interdisciplinary perspective to explain the instability of K_2_CO_3_ catalysts in liquid-phase reactions. Industrial case studies by Kale et al. [20] revealed a 12–15% increase in annual cost for K_2_CO_3_-driven biodiesel plants, while Vazquez-Garrido et al. [21] quantified a 22% decline in the fatty acid methyl ester (FAME) yield after five cycles due to potassium leaching. Da Costa et al. [22] corroborated these findings, showing that potassium migration reduces catalyst surface area from 125 m^2^/g to 78 m^2^/g under high-temperature conditions.

Sodium-based catalysts, however, demonstrate superior stability. Bernard et al. [18] demonstrated that hydrotalcite phase formation in MgO/Na_2_CO_3_ composites effectively prevented structural degradation during cycling, with negligible sodium loss (<0.3 wt%) observed over 10 cycles, while Emeji et al. [23] reported an optimized regeneration efficiency reaching 92% for sodium-based systems, significantly higher than their potassium-based counterparts. Visioli et al. [14] observed a 62% reduction in carbon deposition on γ-Al_2_O_3_/Na systems.

#### 2.5.3. Homogeneous vs. Heterogeneous Catalytic Systems

Homogeneous catalysts, such as K_3_PO_4_ and Na_2_CO_3_, exhibit high initial FAME yields (up to 96% [24]). Malins [24] highlighted the solubility-driven limitations of K_2_CO_3_ (>1.2 g/mL in methanol), which necessitate costly posttreatment processes. In contrast, heterogeneous systems address these challenges through physical confinement and electrostatic stabilization. Santamaría et al. [25] revealed that Mg-doped layered structures (0.53 nm lattice spacing) in sodium-ion batteries analogously stabilize Na^+^ in MgO/Na_2_CO_3_ catalysts, reducing ion leaching. He et al. [26] further demonstrated that sodium’s spatial site-blocking effect lowers glycerol adsorption energy to −1.2 eV, effectively suppressing deactivation. Molecular dynamics simulations by Rostamizadeh et al. [11] attributed sodium’s stability to its thicker solvation layer (0.38 nm vs. 0.28 nm for K^+^), which delays active site coverage by reactants. Table 3 presents a comparison of the performance of various heterogeneous catalysts in biodiesel production.

#### 2.5.4. Research Gaps and Innovation

While existing studies validate the advantages of MgO/carbonate composites, the mechanistic differences between Na^+^ and K^+^ in modulating catalyst recyclability remain underexplored. The current literature lacks systematic analyses of ion-specific effects on interfacial dynamics and long-term stability. This study bridges this gap by integrating in situ spectroscopic characterization and multiscale simulations to unravel Na^+^/K^+^ differential behaviors. Our work pioneers the correlation between ion solvation structures and catalyst deactivation pathways, offering a blueprint for designing next-generation solid base catalysts with industrial viability.

In future research regarding surface engineering, it is necessary to pay attention to the development of gradient calcination technology to reduce sodium migration barriers [9,24]. In the study of advanced characterization, attention should be paid to the use of operand X-ray absorption spectroscopy to track the ion migration at the reaction interface [15,27]. Facing the practicability of economic modeling, it is necessary to establish a life prediction framework to quantify the cost–benefit ratio of large-scale applications [13,28]. By addressing these challenges, this research aims to accelerate the transition from laboratory-scale innovation to sustainable industrial biodiesel production.

### 2.6. Specific Surface Area/Pore Structure, Alkaline Sites, Ionic Dissolution and Stability, Industrial Scale-Up Prospects

#### 2.6.1. Solubility Differences in Methanol and Methanol/Glycerol Systems

**Figure 16:**
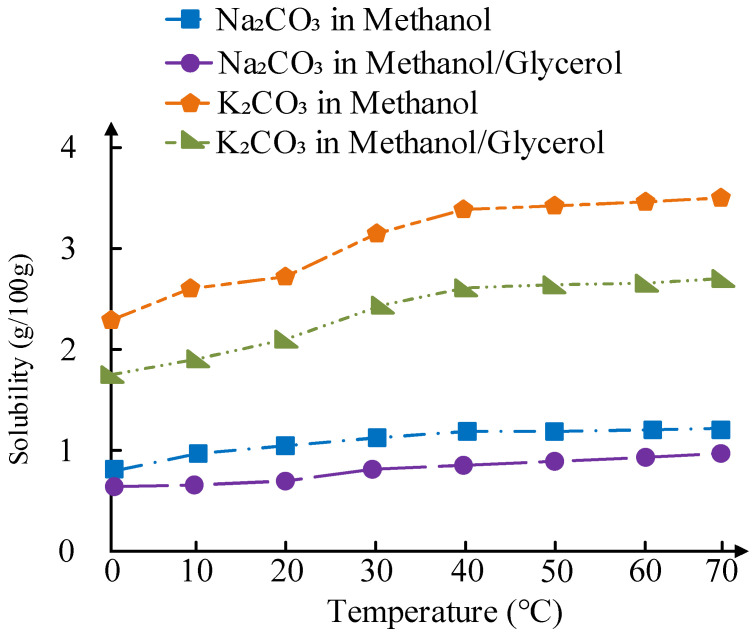
Temperature-dependent solubility of Na_2_CO_3_ and K_2_CO_3_ in methanol and methanol/glycerol (3:1 *v*/*v*).

**Figure 17:**
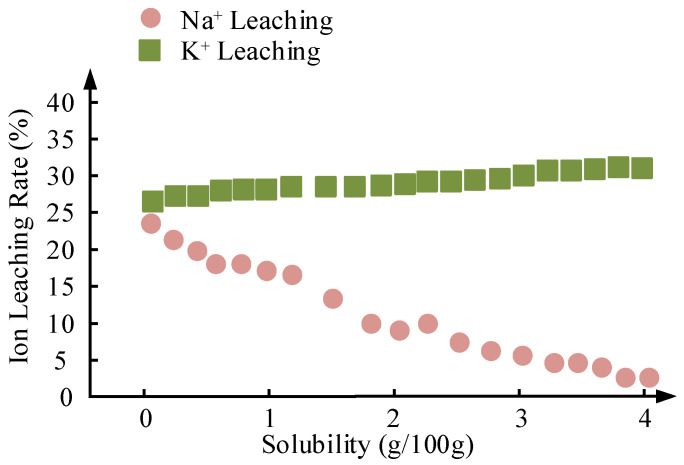
Correlation between solubility and cumulative ion leaching rates (Na^+^ vs. K^+^) after 5 cycles.

The solubility of Na_2_CO_3_ and K_2_CO_3_ in methanol and methanol/glycerol mixtures plays a critical role in active component leaching. As illustrated in Figure 16, the solubility of Na_2_CO_3_ in methanol increases from 0.85 g/100 g at 30 °C to 1.30 g/100 g at 70 °C, while in methanol/glycerol (3:1 *v*/*v*), it ranges from 0.72 g/100 g to 1.04 g/100 g over the same temperature range. In contrast, K_2_CO_3_ exhibits significantly higher solubility: 3.25 g/100 g in methanol at 60 °C and 2.30 g/100 g in methanol/glycerol. This observation aligns with the differences in ion leaching rates among the catalysts. As shown in Figure 17, there is a clear correlation between solubility and the cumulative ion leaching rates after five cycles.

#### 2.6.2. Reusability and Stability Analysis

MgO/Na_2_CO_3_ demonstrated a higher initial FAME yield of 97.5% compared to 95.8% for MgO/K_2_CO_3_. This high initial activity aligns with the literature reports, where Na_2_CO_3_-based catalysts achieved FAME yields exceeding 95% under comparable conditions, confirming the inherent catalytic potential of carbonate species [24]. However, MgO/Na_2_CO_3_ experienced a more pronounced deactivation after seven reaction cycles, retaining 78.5% of its initial yield versus 48.9% for MgO/K_2_CO_3_. This performance decline for MgO/Na_2_CO_3_ primarily resulted from cumulative Na^+^ leaching, measured at 18.9% after five cycles, and significant carbon deposition (22.3 wt%). The leached Na^+^ ions formed insoluble metal soaps (Na-OOCR) within the catalyst pores, leading to pore blockage and a consequent reduction in accessible surface area. In contrast, MgO/K_2_CO_3_, despite exhibiting higher inherent solubility, maintained better structural integrity over cycles due to its denser morphology, which effectively mitigated pore blockage and preserved the surface area. The superior crystalline phase stability of K_2_CO_3_, evidenced by only 12% grain growth compared to 28% for Na_2_CO_3_, further contributed to the enhanced cycling stability of MgO/K_2_CO_3_ relative to its sodium counterpart. Nevertheless, MgO/K_2_CO_3_ still suffered a substantial 46.9% activity loss over seven cycles. This rapid decay stemmed from irreversible carbon deposition (16.8 wt%) and gradual structural collapse induced by prolonged reaction stress. Comparing cycle stability with other MgO-based systems highlights the context; while catalysts like Majedi et al.’s magnetic nanoparticle-supported system showed deactivation within five cycles [12], and Visioli et al.’s γ-Al_2_O_3_ required continuous operation modes [14], the present MgO/carbonate catalysts, particularly MgO/Na_2_CO_3_, demonstrate competitive stability over seven batch cycles, though challenges with leaching and coking remain significant hurdles common to solid base catalysts [23,24].

#### 2.6.3. Regeneration Methods and Performance Recovery

Catalyst deactivation, primarily caused by leaching and carbon accumulation, necessitates effective regeneration strategies. Between each reaction cycle in the stability test, spent catalysts were recovered by filtration, thoroughly washed with ethanol to remove residual reactants and products, and dried at 110 °C for 6 h prior to reuse without intermediate calcination. To systematically address deactivation after multiple cycles, various dedicated regeneration methods were evaluated for their ability to restore performance, with the choice of strategy significantly impacting both recovery efficiency and operational feasibility (Table 4).

The divergent regeneration behavior between MgO/Na_2_CO_3_ and MgO/K_2_CO_3_ stems from inherent material properties. MgO/K_2_CO_3_ exhibits a higher yield recovery, primarily due to the thermal stability and crystallinity of the K_2_CO_3_ phase, facilitating effective recrystallization during thermal treatments like calcination. Its lower hydroxyl density also reduces carbonaceous deposit formation, making deposits more combustible. Conversely, while MgO/Na_2_CO_3_ demonstrates superior cycling stability owing to slower Na^+^ leaching, its regeneration efficiency is hampered by the lower melting point and propensity for the sintering of Na_2_CO_3_ components during high-temperature regeneration. This can lead to irreversible agglomeration, the partial redistribution of active sodium species, and reduced accessible surface area post-regeneration [24].

High-temperature calcination at 600 °C for 2 h proved effective for carbon removal, achieving a 95.7% removal rate and 78.4% active site recovery for MgO/Na_2_CO_3_. However, this method induced significant grain growth, limiting the surface area recovery to 88.5%. Plasma treatment under an argon atmosphere emerged as the most effective method for MgO/K_2_CO_3_, yielding a 93.5% FAME yield recovery and 97.3% carbon removal, albeit at a high energy cost of 24.7 MJ/kg and high operational complexity. Supercritical CO_2_ treatment offered a more environmentally friendly alternative under mild conditions (31 °C, 7.4 MPa), achieving a moderate 75.6% yield recovery for MgO/Na_2_CO_3_, but its industrial implementation is constrained by specialized equipment requirements and cost. Acid washing with 0.1 M HCl, while energy-efficient (8.2 MJ/kg), caused structural damage, evident in only 72.3% surface area recovery for MgO/Na_2_CO_3_, and risked Cl^−^ contamination, rendering it unsuitable for sustained use. Ultrasonic cleaning showed limited efficacy across all metrics, and enzyme cleaning, despite its eco-friendly profile, yielded the lowest recovery rates (<62%), underscoring the persistent challenge in balancing sustainability with regeneration efficacy. Chemical reduction with H_2_ at 400 °C provided a moderate recovery but involved significant energy consumption and complexity. In practical applications, calcination remains the most common industrial regeneration method for solid catalysts due to its operational simplicity and effectiveness against carbon deposits [14,24], despite its energy demands and potential for sintering. Plasma treatment shows exceptional promise for performance recovery but requires substantial cost reductions for wider adoption. The selection hinges critically on weighing the required recovery level against energy expenditure, equipment costs, and process complexity for the specific catalyst and intended scale of operation.

Assessing industrial feasibility requires balancing recovery efficiency, energy consumption, equipment cost, and operational complexity. High-temperature calcination remains the most practical large-scale method due to its operational simplicity, established infrastructure, and effectiveness against carbon deposits, despite significant energy demands (~12.4 MJ/kg) and risks of sintering. Plasma treatment offers superior performance recovery (up to 93.5% yield recovery, 97.3% carbon removal) but is currently prohibitive for industrial scaling due to very high energy costs (~24.7 MJ/kg) and complex reactor requirements. Supercritical CO_2_ provides an environmentally milder alternative but faces challenges with specialized high-pressure equipment costs and moderate recovery rates (~75.6%). Acid washing, while energy-efficient (~8.2 MJ/kg), causes structural degradation and contamination risks, limiting its sustainability. For large-scale biodiesel plants prioritizing operational robustness and cost-effectiveness, optimized calcination protocols represent the most viable current regeneration strategy, though plasma technology warrants further development for cost reduction [14,24].

#### 2.6.4. Comparative Analysis of Catalyst Performance

The divergent performance between MgO/Na_2_CO_3_ and MgO/K_2_CO_3_ catalysts arises from the interplay of their physicochemical properties and reaction mechanisms, as detailed in Table 5.

The significantly higher initial activity of MgO/Na_2_CO_3_ (97.5% FAME yield) versus MgO/K_2_CO_3_ (95.8%) is mechanistically attributed to three synergistic structural advantages derived from comprehensive characterization:

(1). Enhanced Basicity and Uniform Site Distribution:

Na^+^ (0.95 Å) incorporation into MgO lattice (XRD peak shift: 36.9° → 37.0°; lattice constant reduction: 4.209 Å) generates oxygen vacancies that increase electron density, creating strongly basic sites (CO_2_-TPD peak at 265 °C vs. 447 °C for K_2_CO_3_) [17]. EDS mapping confirms superior Na homogeneity (uniformity index: 0.92) versus K^+^ (0.87), enabling efficient nucleophilic attack on triglycerides.

(2). Optimized Porosity for Mass Transfer:

BET analysis reveals MgO/Na_2_CO_3_ possesses 9.8% higher surface area (148.6 m^2^/g) and 20% larger pore volume (0.42 cm^3^/g) than MgO/K_2_CO_3_. Its homogeneous mesopores (Figure 1b,f) facilitate reactant diffusion, while K^+^-induced pore blockage (126.3 m^2^/g, Table 1) restricts access to active sites.

(3). Thermal Stability-Controlled Crystallinity:

Calcination at 600 °C yields an ideal MgO crystallite size (9.8 nm) with fully open pores. Na_2_CO_3_’s lower decomposition temperature (380 °C vs. 420 °C for K_2_CO_3_) promotes amorphous phase formation, increasing accessible basic sites. K_2_CO_3_’s crystalline rigidity reduces active site density despite superior thermal stability [24,29].

These structural advantages collectively explain the 1.7% higher initial FAME yield of MgO/Na_2_CO_3_. The smaller Na^+^ radius enables deeper lattice integration, forming stable Na-O-Mg bonds that optimize both basicity and porosity—whereas K^+^’s larger size (1.33 Å) limits subsurface diffusion, causing surface enrichment and rapid deactivation via leaching/coking [27,30].

MgO/Na_2_CO_3_ delivered a superior initial FAME yield of 97.5% compared to 95.8% for MgO/K_2_CO_3_, attributable to its higher specific surface area (148.6 m^2^/g versus 126.3 m^2^/g) and greater alkaline site density. The smaller ionic radius of Na^+^ (0.95 Å) facilitates its integration into the MgO lattice, forming stable Na-O-Mg bonds and inducing oxygen vacancies. These vacancies enhance electron density, creating strongly basic sites evidenced by a CO_2_-TPD high-temperature peak at 265 °C, contrasting with the 447 °C peak observed for K_2_CO_3_. Furthermore, the mesoporous structure of MgO/Na_2_CO_3_ promotes efficient reactant diffusion and minimizes glycerol accumulation (5.2% versus 6.8% for K_2_CO_3_).

Despite its higher initial activity, MgO/Na_2_CO_3_ exhibited a more significant decline in performance over cycles. After five reaction cycles, its FAME yield decreased to 88.2%, while MgO/K_2_CO_3_ retained only 65.2% of its initial yield. This greater relative stability for the sodium-based catalyst occurs despite substantial cumulative Na^+^ leaching (18.9%) and carbon deposition (22.3 wt%), which caused severe pore blockage, reducing its specific surface area from 148.6 m^2^/g to 61.2 m^2^/g. MgO/K_2_CO_3_, benefiting from a denser crystalline structure that mitigated pore blockage, still suffered from higher inherent solubility in methanol (3.25 g/100 g at 60 °C versus 1.15 g/100 g for Na_2_CO_3_), leading to a significant loss of active components. The rapid activity decay observed in MgO/K_2_CO_3_ aligns with challenges reported for carbonate-based catalysts facing irreversible carbon deposition and structural fatigue under prolonged reaction conditions [24,26].

The post-regeneration analysis revealed a critical difference in recoverability. MgO/K_2_CO_3_ demonstrated a higher yield recovery rate of 94.2% compared to 88.5% for MgO/Na_2_CO_3_. This superior regeneration efficiency for the potassium-based system stems from the inherent structural stability of the K_2_CO_3_ crystalline phase. This stability facilitates the effective restoration of the active phase during thermal regeneration processes like calcination [23]. In contrast, the partial solubility of Na_2_CO_3_ components during regeneration may lead to irreversible redistribution or the loss of active sodium species [24], limiting the recovery potential of MgO/Na_2_CO_3_. K_2_CO_3_ also demonstrated higher thermal stability (decomposition temperature 420 °C versus 380 °C for Na_2_CO_3_) and retained more surface area (91.7% versus 82.4%) post-regeneration, minimizing structural damage. Additionally, FTIR indicated a 32% reduction in hydroxyl peak intensity for K_2_CO_3_, suggesting enhanced surface hydrophobicity that suppresses glycerol adsorption and reduces carbon deposition rates (3.2% versus 4.1% for Na_2_CO_3_), further aiding regeneration efficacy.

Economically, MgO/Na_2_CO_3_ presents a lower feedstock cost (1200 USD/ton versus 1350 USD/ton) due to the abundance and lower market price of sodium carbonate, consistent with the literature indicating sodium salts can be approximately 40% cheaper than potassium salts [24]. However, MgO/K_2_CO_3_’s higher regeneration recovery rate and potentially longer effective operational life in processes demanding frequent catalyst regeneration, as seen in continuous systems [21,23], could offset its higher initial material cost over time. Strategies to improve both systems exist; doping MgO/Na_2_CO_3_ with Al_2_O_3_ (5 wt%) or applying a SiO_2_ core-shell coating could suppress grain overgrowth and pore collapse during high-temperature regeneration. For MgO/K_2_CO_3_, enhancing mass transfer efficiency through hierarchical pore design could compensate for its slightly lower initial activity. Addressing carbon deposition and active phase redistribution remains crucial for enhancing the long-term viability of both catalyst types in industrial biodiesel production [8,23,24].

## 3. Materials and Methods

The optimization of solid alkali catalysts should be based on the systematic regulation of their physicochemical properties and in-depth analysis of their reaction mechanisms. In this study, magnesium oxide was used as the carrier, and the porous composite catalysts were constructed through the differential loading of sodium carbonate and potassium carbonate, which was combined with the synergistic optimization of the calcination temperature, the alcohol–oil ratio, and the loading amount to explore the influence of sodium and potassium ions on the structure of the catalysts and the efficiency of the ester exchange. The experimental design covered catalyst preparation, physicochemical characterization (specific surface area, pore structure, basic site distribution), and reaction performance testing (biodiesel yield, cyclic stability). All the reagents were of analytical purity, and the key instrumental parameters were strictly calibrated to ensure the reliability of the data. The following sections describe in detail the implementation of the experimental materials, methods, and analytical techniques.

### 3.1. Experimental Reagents and Instruments

(1) Experimental reagents

The reagents used in the experiments are shown in Table 6; all chemical reagents were analytically pure (AR) and the soybean oil was of first-class edible grade (Nissin Salad Oil Co., Suzhou, China) and was not pretreated before use.

### 3.2. Experimental Methods

(1) Catalyst preparation steps

The MgO-supported Na_2_CO_3_/K_2_CO_3_ composite catalysts were synthesized via incipient wetness impregnation using analytically pure reagents (Table 6) without pretreatment. Anhydrous sodium carbonate or potassium carbonate was dissolved in deionized water to prepare 0.20 mol/L solutions. To systematically investigate the effects of loading and calcination temperature, catalysts were prepared with Na_2_CO_3_/K_2_CO_3_ loadings of 10, 20, 30, 35, 40, and 50 wt% relative to MgO, and calcined at 500, 550, 600, 650, or 700 °C under nitrogen flow. For a representative 35% mass loading relative to MgO (3.00 g), the required carbonate solution volume was mixed with MgO powder under stirring for 3 h. The slurry was dried at 120 °C for 10 h, ground, and calcined (5 °C/min ramp) for 3 h. All catalysts follow the following naming convention: X%-Na_2_CO_3_/MgO-Y °C, X%-K_2_CO_3_/MgO-Y °C and their abbreviated forms X-Na/Mg-Y, X-K/Mg-Y, where X = carbonate loading (wt%), Na/K = carbonate type, Mg = MgO support, and Y = calcination temperature (°C).

(2) Transesterification Reaction

**Figure 18:**
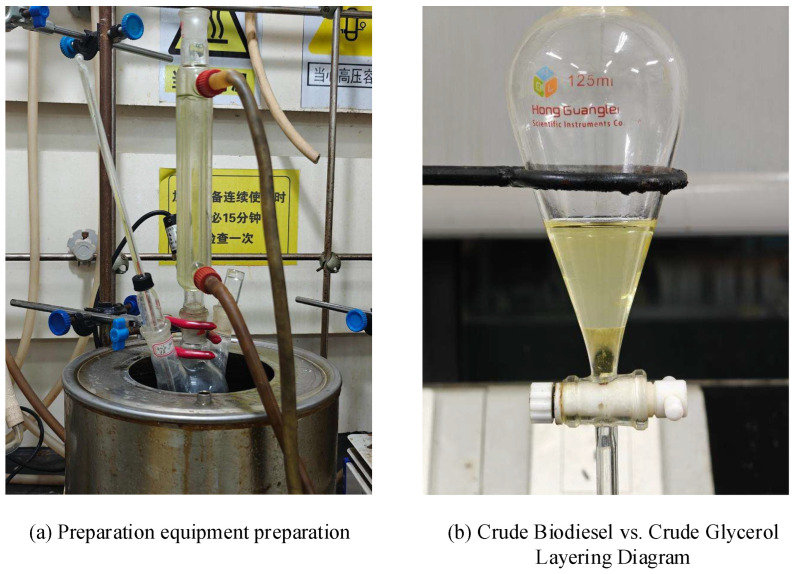
Experimental preparation and results of biodiesel preparation via ester exchange reaction. Note: In subfigure (**a**), the text accompanying the instrument states: “When the heating equipment is used continuously, it must be inspected every 15 min. Beware of high-pressure vessels.”

Soybean oil and methanol (12:1 molar ratio) were reacted at 65 °C with mechanical stirring (500× rpm) for 3 h using 3.0 wt% catalyst (e.g., 0.48 g catalyst per 16.0 g oil). It is important to highlight that all reaction conditions were systematically fine-tuned in subsequent steps. Herein, only the specific reaction conditions are presented as representative examples for illustration purposes. Post-reaction, the catalyst was filtered, washed with methanol, and dried for stability analysis. The product mixture underwent low-pressure distillation to remove excess methanol, followed by phase separation in a separatory funnel to isolate crude biodiesel and glycerol [27,28,29,31,32,33,34,35,36]. Glycerol concentrations were adjusted based on experimental yields (e.g., 0.010–0.005 g/mL) for spectrophotometric quantification (Supplementary Materials Figure S1) to further determine the yield of FAME. The experimental preparation and liquid separation processes for biodiesel obtained through the transesterification reaction are illustrated in Figure 18.

(3) Reagent Storage

Soybean oil (first-grade edible) and methanol were stored in sealed containers to minimize moisture interference during reactions.

(4) Transesterification reaction and biodiesel analysis

In this study, a novel analytical method—the copper glycerol standard colorimetric assay—was utilized to quantify biodiesel yield through the measurement of glycerol generated during the transesterification reaction. Further details regarding the specific operation steps, FAME yield calculation method, method limitations, and experimental apparatus are provided in the Supplementary Materials.

### 3.3. Quality Control and Safety Protocols

To ensure the reliability of the experimental data and the safety of the operation process, multi-level quality control measures and safety regulations were implemented in this research system [37,38,39,40,41]. During the catalyst preparation stage, all batches of MgO-based composites were subjected to X-ray diffraction (XRD, Rigaku SmartLab, Cu-Kα radiation, Osaka, Japan) to verify the consistency of the crystal structure, with the deviation of the diffraction peak intensities controlled to be within 5% and compared with a standard JCPDS card (JCPDS 45-0946) to ensure that no heterogeneous phases were generated. The specific surface area and pore size distribution were regularly assessed by means of the nitrogen adsorption–desorption method (BET, Micromeritics ASAP 2460, Norcross, GA, USA), and 1 sample was randomly selected for testing in every 5 batches, requiring the fluctuation range of the specific surface area to be less than ±3 m^2^/g, in order to maintain the stability of the catalyst’s physical properties. For the high-temperature calcination process, the tube furnace (OTF-1200X-S, Hefei Kejing Material Technology, Hefei, China) required a 10 min nitrogen pre-purge before each run to ensure that the oxygen content in the chamber was less than 50 ppm, while the operator was required to wear high-temperature-resistant gloves (up to 1200 °C) and goggles to prevent thermal radiation injury.

In the transesterification reaction stage, the storage and use of methanol strictly followed the ‘Regulations on the Safe Management of Hazardous Chemicals’, and all operations involving methanol were carried out in a special fume hood equipped with an explosion-proof ventilation system (air velocity ≥ 0.5 m/s), and the experimental personnel were required to wear a class A gas mask (3M 6800, with an organic vapor filter cartridge Minnesota Mining and Manufacturing Company, Saint Paul, MN, USA) and chemical-resistant gloves (made from nitrile material, with a thickness of 0.5 mm). The pressure control in the reaction system was monitored in real time by the pressure relief valve of the autoclave (GSH-20), and the safety threshold was set at 8 MPa, which automatically triggered the shutdown protection in case of overpressure. In order to reduce the influence of catalyst loss on the data, each batch of catalyst after reaction was subjected to a standardized recovery process of centrifuged at 8000× rpm(10 min) and methanol washing (3 times, 50 mL each), and the mass loss rate was recorded by an electronic balance (MX204, Mettler Toledo, HongKong, China), requiring that the mass loss in a single cycle should not exceed 2% of the initial value.

For data reliability control, three independent replications were set for all experimental conditions; the experimental results were presented in the form of mean ± standard deviation (SD), and significant outliers were identified and rejected according to Grubbs’ test (95% confidence level) [41,42,43,44,45,46]. The UV–visible spectrophotometer (UV-1800, Shanghai Yiheng Instrument, Shanghai, China) used for the determination of glycerol fat content was calibrated by a standard praseodymium–neodymium filter for wavelength calibration every day before start-up to ensure that the measurement error of absorbance was less than ±0.002. The original data were stored using a double back-up mechanism, and the plots, spectra, and calculation tables generated in the course of the experiments were uploaded to the encrypted server of the laboratory (with IP address restriction on the access) and the offline hard disk to ensure the traceability of the data. The hard disk ensured the traceability of the data.

For experimental waste treatment, waste methanol and biodiesel crude products were collected in special explosion-proof containers (made of 316L stainless steel) and entrusted to qualified third-party environmental protection companies (e.g., China Energy Conservation and Environmental Protection Group) to carry out high-temperature incineration (1200 °C, residence time ≥ 2 s) to ensure that the decomposition rate of dioxin and other hazardous substances exceeded 99.9%. The laboratory is equipped with an emergency sprinkler system (response time < 1 s) and acid and alkali neutralization tanks (pH range 4–10) to cope with emergencies such as chemical leakage [30,47,48,49,50].

Through the comprehensive measures outlined above, this study effectively minimized experimental risks while simultaneously enhancing the credibility of the collected data.

## 4. Conclusions

This study demonstrates that MgO/Na_2_CO_3_ and MgO/K_2_CO_3_ solid base catalysts serve as viable alternatives to homogeneous catalysts, exhibiting distinct trade-offs in activity and stability. The optimal reaction conditions for the MgO/Na_2_CO_3_ alkaline catalyst are as follows: 65 °C, 4.0 wt% loading, 12:1 methanol-to-oil ratio, and 3 h. The MgO/Na_2_CO_3_-600 °C catalyst achieved a high initial FAME yield of 97.5%, owing to its mesoporous structure and strong basicity. However, its performance declined to 88.2% after five cycles due to Na^+^ leaching (18.9%) and carbon deposition (22.3 wt%). The MgO/K_2_CO_3_-600 °C catalyst displayed a FAME yield that decreased to 65.2% after five cycles and a slightly lower initial yield of 95.8%, attributed to its high K^+^ leaching (27.7%) and carbon accumulation (16.8 wt%), leading to an accelerated rate of catalyst deactivation.

Mechanistic investigations revealed that the smaller ionic radius of Na^+^ (0.95 Å) enabled lattice embedding for uniform active site distribution, while K^+^ surface enrichment enhanced framework stability. The superior thermal stability of K_2_CO_3_, evidenced by a 40 °C higher decomposition temperature (420 °C vs. 380 °C for Na_2_CO_3_), combined with its hydrophobic properties (32% reduction in surface hydroxyl groups), significantly improved resistance to degradation during regeneration. Economically, although MgO/Na_2_CO_3_ exhibited an 11% lower feedstock cost (USD 1200/ton vs. USD 1350/ton), MgO/K_2_CO_3_ demonstrated greater long-term cost-effectiveness through higher regeneration efficiency (94.2% vs. 88.5%). Overall, although sodium-based alkaline catalysts have higher activity and reusability, considering the cost of recovery, the current heterogeneous solid catalysts still mainly choose potassium salts as their main components.

To address technical limitations, strategic modifications such as Al_2_O_3_ doping or SiO_2_ core–shell architectures are proposed to mitigate pore collapse in Na_2_CO_3_-based catalysts, while hierarchical pore engineering could enhance mass transfer in K_2_CO_3_ systems. Life cycle assessments are recommended to quantify environmental benefits and guide industrial implementation. By elucidating the critical role of alkali metal ion properties in balancing catalytic activity and durability, this research provides theoretical foundations and practical insights for advancing sustainable biodiesel production technologies.

## Figures and Tables

**Table 1:** BET surface area, pore volume, and average pore diameter of catalysts.

Catalyst	Surface Area (m^2^/g)	Total Pore Volume (cm^3^/g)	Average Pore Diameter (nm)
MgO-600 °C	105.4 ± 3.5	0.51 ± 0.02	9.8 ± 0.3
35%-Na_2_CO_3_/MgO-600 °C	148.6 ± 3.2	0.42 ± 0.02	8.7 ± 0.3
35%-K_2_CO_3_/MgO-600 °C	126.3 ± 2.8	0.35 ± 0.01	9.2 ± 0.4
35%-Na_2_CO_3_/MgO-500 °C	112.4 ± 2.5	0.28 ± 0.01	7.9 ± 0.2
35%-Na_2_CO_3_/MgO-700 °C	98.5 ± 1.9	0.21 ± 0.01	6.5 ± 0.3

**Table 2:** Catalyst matrix for systematic optimization.

Sample Code	Loading (wt%)	Calcination (°C)	Carbonate	Test Scope
10-Na/Mg-600	10	600	Na_2_CO_3_	Loading screening
20-Na/Mg-600	20	600	Na_2_CO_3_	Loading screening
30-Na/Mg-600	30	600	Na_2_CO_3_	Loading screening
35-Na/Mg-600	35	600	Na_2_CO_3_	All tests
40-Na/Mg-600	40	600	Na_2_CO_3_	Loading screening
50-Na/Mg-600	50	600	Na_2_CO_3_	Loading screening
35-Na/Mg-500	35	500	Na_2_CO_3_	Temperature effect
35-Na/Mg-550	35	550	Na_2_CO_3_	Temperature effect
35-Na/Mg-650	35	650	Na_2_CO_3_	Temperature effect
35-Na/Mg-700	35	700	Na_2_CO_3_	Temperature effect
35-K/Mg-600	35	600	K_2_CO_3_	Ion comparison

**Table 3:** Comparative performance of heterogeneous catalysts in biodiesel production.

Catalyst Type	Surface Area (m^2^/g)	FAME Yield (%)	Cycle Stability (5 Cycles)	Reference
Fe-Al-O (Al^3+^-doped)	85	89	18% activity drop	[17]
Na-montmorillonite	112	93	0.8 wt% Na loss	[10]
Mo/ZIF-8	980	97	91% retention	[11]
γ-Al_2_O_3_/Na	125 → 78 *	91 → 72 *	62% less coking	[14]
MgO/Na_2_CO_3_	145	95	88% retention	[25]

Note: * Post-reaction values under high-temperature conditions. “→” Representing the variation in numerical values.

**Table 4:** Recovery effects of different regeneration methods on catalyst performance.

Regeneration Method	MgO/Na_2_CO_3_ Yield Recovery (%)	MgO/K_2_CO_3_ Yield Recovery (%)	Surface Area Recovery (%)	Carbon Removal Rate (%)	Active Site Density Recovery (%)	Energy Consumption (MJ/kg)	Operational Complexity (1–5)
Calcination (600 °C, 2 h)	82.3 ± 1.5	91.2 ± 1.2	88.5 ± 2.0	95.7 ± 1.8	78.4 ± 2.5	12.4 ± 0.8	2
Acid Washing (HCl, 0.1 M)	65.7 ± 2.0	73.8 ± 1.8	72.3 ± 2.5	85.2 ± 2.3	61.2 ± 3.0	8.2 ± 0.5	4
Ultrasonic Cleaning (40 kHz)	58.9 ± 2.5	67.5 ± 2.1	65.8 ± 3.0	76.4 ± 2.8	53.4 ± 3.5	5.6 ± 0.3	3
Supercritical CO_2_ Treatment	75.6 ± 1.8	84.3 ± 1.5	80.1 ± 2.3	90.1 ± 1.9	70.8 ± 2.8	18.9 ± 1.2	5
Plasma Treatment (Ar)	88.9 ± 1.2	93.5 ± 1.0	92.7 ± 1.5	97.3 ± 0.9	85.3 ± 2.0	24.7 ± 1.5	5
Chemical Reduction (H_2_, 400 °C)	71.2 ± 1.8	79.6 ± 1.6	76.3 ± 2.1	88.5 ± 2.0	68.9 ± 2.5	15.3 ± 1.0	4
Enzyme Cleaning	53.4 ± 3.0	62.1 ± 2.5	60.2 ± 3.2	70.3 ± 3.5	49.7 ± 3.8	3.8 ± 0.2	3

**Table 5:** Comparison of comprehensive performance of MgO/Na_2_CO_3_ and MgO/K_2_CO_3_ catalysts.

Parameter	35%-Na_2_CO_3_/MgO-600 °C	Key Points of Explanation	35%-K_2_CO_3_/MgO-600 °C	Key Points of Explanation	Comparison of Regeneration Advantages
Initial Yield (%)	97.5 ± 0.1	High specific surface area (148.6 m^2^/g)+strong alkali site density (CO_2_-TPD 265 °C)	95.8 ± 0.2	Lower specific surface area (126.3 m^2^/g)+weak base site (CO_2_-TPD 447 °C)	/
Yield after 5th Cycle (%)	88.2 ± 0.5	Na^+^ dissolution is slow (18.9%) but carbon deposition is serious (22.3 wt%) → pore blockage	65.2 ± 0.8	High K^+^ dissolution (27.7%) + structural collapse → rapid loss of active sites	Better cycle stability of Na system
Specific Surface Area (m^2^/g)	148.6 ± 3.2	Na^+^ embedded in MgO lattice → homogeneous mesoporous	126.3 ± 2.8	K^+^ surface enrichment → pore blockage	/
Na^+^/K^+^ Leaching Rate (%)	18.9 ± 0.1	Low solubility of Na_2_CO_3_ methanol (1.15 g/100 g)	27.7 ± 0.1	High solubility of K_2_CO_3_ (3.25 g/100 g)	Better dissolution control of Na system
Yield Recovery after Regeneration (%)	88.5 ± 1.5	Na_2_CO_3_ is easy to sinter during regeneration → irreversible loss of active site	94.2 ± 1.3	K_2_CO_3_ crystal phase is stable + carbon deposit is easy to remove → active phase is recovered efficiently	Higher regeneration recovery rate of K series
Economic Cost (USD/ton)	1200	Na_2_CO_3_ raw material is cheap (40% lower than K_2_CO_3_ [17])	1350	The price of K_2_CO_3_ is higher	Na series is economical

**Table 6:** List of experimental reagents.

Reagent Name	Manufacturer	CAS Number	Concentration/Purity
Light Magnesium Oxide	Sinopharm Chemical Reagent Co., Ltd. Shanghai, China.	1309-48-4	Analytical Grade/AR
Anhydrous Sodium Carbonate	Sinopharm Chemical Reagent Co., Ltd. Shanghai, China	497-19-8	Analytical Grade/AR
Anhydrous Potassium Carbonate	Sinopharm Chemical Reagent Co., Ltd. Shanghai, China.	584-08-7	Analytical Grade/AR
Glycerol	Sinopharm Chemical Reagent Co., Ltd. Shanghai, China.	56-81-5	Analytical Grade/AR
Anhydrous Methanol	Sinopharm Chemical Reagent Co., Ltd. Shanghai, China.	67-56-1	Analytical Grade/AR
Soybean Oil	Nisshin Seifun Group, Inc., Suzhou, China.	N/A	First-Grade Edible Soybean Oil

## Data Availability

The original contributions presented in this study are included in the article/Supplementary Material. Further inquiries can be directed to the corresponding author.

## Supplementary Materials

The following are available online at https://www.mdpi.com/article/10.3390/molecules30132876/s1. References [51,52,53] are cited in the supplementary materials.

## Abbreviations

The following abbreviations are used in this manuscript:

FAME	Fatty Acid Methyl Ester
SEM	Scanning Electron Microscope
EDS	Energy Dispersive Spectroscopy
BET	Brunauer–Emmett–Teller
TPD	Temperature Programmed Desorption
FTIR	Fourier Transform Infrared spectroscopy
TG	Thermogravimetry Analysis
DTG	Derivative Thermogravimetry Analysis

## References

[1] Spanou A., Liakouli N.C., Fiotaki C., Pavlidis I.V. (2024). Comparative Study of Immobilized Biolipasa-R for Second Generation Biodiesel Production from an Acid Oil. ChemBioChem.

[2] Hoe B.C., Arumugam P., Chew I.M.L., Tan J., Ooi C.W. (2024). Extraction of Palm Carotene from Crude Palm Oil by Solvolytic Micellization: Economic Evaluation and Life Cycle Assessment. Chem. Eng. Commun..

[3] Colaco L.A., Sousa A.S., Costa A.C.F.M., Farias A.F.F., Santos I.M.G. (2024). Zinc Molybdate: A New Catalyst For Biodiesel Synthesis By Simultaneous Esterification/Transesterification Reaction. Fuel.

[4] Kim S.E., Kim J.H., Kim D.K., Ham H.C., Lee K.Y., Kim H.J. (2023). Na-Modified Carbon Nitride as a Leach-Resistant and Cost-Effective Solid Base Catalyst for Biodiesel Production. Fuel.

[5] Winfield D.M.D., Cermak S.C., Evangelista R.L., Moser B.R., McKinney J., Pantalone V. (2024). Evaluation of a High Oleic Soybean Oil Variety in Lubricant and Biodiesel Applications. J. Am. Oil Chem. Soc..

[6] Agbulut U., Sathish T., Kiong T.S., Sambath S., Mahendran G., Kandavalli S.R., Sharma P., Gunasekar T., Kumar P.S., Saravanan R. (2024). Production of Waste Soybean Oil Biodiesel with Various Catalysts, and the Catalyst Role on The CI Engine Behaviors. Energy.

[7] Pariyan K., Ahmadi A., Hosseini M.R. (2023). Effect of Thermal Pretreatment on the Two-Stage Extraction of Aluminum, Sulfur and Potassium from A Fine-Quartz Bearing Alunite. Miner. Eng..

[8] Sonnemberg M.N.S., Souza E.F., Ventura M., Simionatto E., Fiorucci A.R. (2024). Investigation of Curcumin Antioxidant Efficiency on Oxidation Stability of Biodiesel from Soybean Oil and Beef Tallow, Contaminated with Metals: Kinetic and Storage Studies. Fuel.

[9] Ying A., Bai L., Jiang X., Shen R., Liu Y., Liu Z. (2024). Boosting Catalytic Efficiency of Lipase By Regulating Amphiphilic Microenvironment Through Reversible Addition-Fragmentation Chain Transfer Polymerized Modifications On Polyacrylonitrile Fiber. Int. J. Biol. Macromol..

[10] She Q., Qiu M., Li K., Liu J., Zhou C. (2023). Acidic and Basic Sites on the Surface of Sodium Montmorillonite Active for Catalytic Transesterification of Glycerol to Glycerol Carbonate. Appl. Clay Sci..

[11] Rostamizadeh M., Oghabi M., Ghadimi A. (2023). Highly Efficient and Reusable Mo/ZIF-8 Nanocatalyst In Esterification Reaction For Biodiesel Production. Res. Chem. Intermed..

[12] Majedi M., Safaei E. (2023). Molybdenum (VI) Complex of Resorcinol-Based Ligand Immobilized on Silica-Coated Magnetic Nanoparticles for Biodiesel Production. Appl. Organomet. Chem..

[13] Shao X.B., Liu S., Xing Z.W., Tang J.X., Li P., Liu C., Chi R.Z., Tan P., Sun L.B. (2024). Atomically Dispersed Magnesium with Unusual Catalytic Activity For Transesterification Reaction. AIChE J..

[14] Visioli L.J., Nunes A.L.B., Wancura J.H.C., Enzweiler H., Vernier L.J., Castilhos F. (2023). Batch and Continuous γ-Alumina-Catalyzed FAME Production from Soybean Oil Deodorizer Distillate by Interesterification. Fuel.

[15] Krótki A., Spietz T., Dobras S., Chwola T., Tatarczuk A., Zorawski D., Skowron K., Skrzyniecki D., Hulisz P. (2025). Carbon capture pilot study in Solvay soda ash process. Appl. Energy.

[16] Wang H., Shi Z., Guo K., Wang J., Gong C., Xie X., Xue Z. (2023). Boronic Ester Transesterification Accelerates Ion Conduction for Comb-Like Solid Polymer Electrolytes. Macromolecules.

[17] Ikeue K., Miyamoto Y., Ando E. (2023). Metal-Substituted Layered Fe-Based Oxides as a Solid Base Catalyst. Chem. Phys. Lett..

[18] Bernard E., Yio M., Rentsch D., Chen H., Myers R.J. (2024). Insights on the Effects of Carbonates and Phosphates on the Hydration of Magnesia (Alumino-)Silicate Cements. Appl. Geochem..

[19] Lobo R.R., Arce-Cordero J.A., So S., Soltis M., Marinho M.N., Agustinho B.C., Ravelo A.D., Vinyard J.R., Johnson M.L., Monteiro H.F. (2023). Production, Physiological Response, and Calcium And Magnesium Balance of Lactating Holstein Cows Fed Different Sources of Supplemental Magnesium with or Without Ruminal Buffer. J. Dairy Sci..

[20] Kale B.N., Patle S.D., Kalambe S.R., Khawale V.R. (2024). Comparative Analysis of Compression Ignition Engines Performance and Emission Characteristics Devouring Edible and Nonedible Oil Biodiesel. Environ. Prog. Sustain. Energy.

[21] Vazquez-Garrido I., Guevara-Lara A., Lopez-Benitez A. (2023). Hydroprocessing of New and Waste Soybean Oil for Obtaining Biodiesel: An Operational Conditions Study. Chem. Eng. J..

[22] Da Costa G.B., De Fernandes D.D.S., Veras G., Dias Gondim P.H.G., Gondim A.D. (2024). Combining NIR Spectroscopy with DD-SIMCA for Authentication and iSPA-PLS-DA for Discrimination of Ethyl Route and Oil Feedstocks of Biodiesels in Biodiesel/Diesel Blends. J. Am. Oil Chem. Soc..

[23] Emeji I.C., Patel B. (2024). Box-Behnken Assisted RSM and ANN Modelling for Biodiesel Production over Titanium Supported Zinc-Oxide Catalyst. Energy.

[24] Malins K. (2018). The potential of K_3_PO_4_, K_2_CO_3_, Na_3_PO_4_ and Na_2_CO_3_ as reusable alkaline catalysts for practical application in biodiesel production. Fuel Process. Technol..

[25] Santamaría C., Morales E., Río C., Herradón B., Amarilla J.M. (2023). Studies on Sodium-Ion Batteries: Searching for the Proper Combination of the Cathode Material, the Electrolyte and the Working Voltage. the Role Of Magnesium Substitution in Layered Manganese-Rich Oxides, And Pyrrolidinium Ionic Liquid. Electrochim. Acta.

[26] He P., Zhang W., Fu G., Xie E., Wang W., Zhang Z., Zhang T., Wu G. (2024). Two-Dimensional Mo Catalysts Supported on the External Surface of Planar Silicalite-1 Zeolite for Biodiesel Production from Waste Cooking Soybean Oil: Transesterification Experiment and Kinetics. Fuel.

[27] Hu M., Pu J., Qian E.W., Wang H. (2023). Biodiesel production using MgO–CaO catalysts via transesterification of soybean oil: Effect of MgO addition and insights of catalyst deactivation. BioEnergy Res..

[28] Margellou A.G., Koutsouki A.A., Petrakis D.E., Kontominas M.G., Pomonis P.J. (2022). Catalysis and inhibition of transesterification of rapeseed oil over MgO–CaO. BioEnergy Res..

[29] Xie W., Han Y., Tai S. (2017). Biodiesel Production Using Biguanide-Functionalized Hydroxyapatite-Encapsulated-Gamma-Fe_2_O_3_ Nanoparticles. Fuel.

[30] Zheng J., Tao S., Yang Y., Tang Y. (2024). Gel–sol synthesis of hierarchical CaO using pollen as biotemplate for biodiesel production. Comptes Rendus. Chim..

[31] Yang Z., Liu X., Ma X., Cao T., Xu J., Feng H., Diao R., Qi F., Huang H., Ma P. (2024). Efficient Preparation of Biomass-Based Ultra-Thin 2D Porous Carbon Materials by In Situ Template-Activation and Its Application in Sodium Ion Capacitors. Adv. Funct. Mater..

[32] Liu W., Liu K., McClements D.J., Jin Z., Chen L. (2025). Fabrication and Characterization of Starch-Based Bigels Under Phase Control: Structural, Physicochemical and 3D Printing Properties. Food Hydrocoll..

[33] Zou S., Zhou J., Du Y., Cheng J., Wang Y., Zhang Z. (2023). Texture and Volatile Profiles of Beef Tallow Substitute Produced by a Pilot-Scale Continuous Enzymatic Interesterification. Food Chem..

[34] Tahmasebi-Boldaji R., Rashidi S., Rajabi-Kuyakhi H., Tahmasebi-Boldaji N., Baghdadi M., Karbassi A. (2024). Application of pharmaceutical waste as a heterogeneous catalyst for transesterification of waste cooking oil: Biofuel production and its modeling using predictive tools. Biofuels.

[35] Sajjad N., Orfali R., Perveen S., Rehman S., Sultan A., Akhtar T., Nazir A., Muhammad G., Mehmood T., Ghaffar S. (2022). Biodiesel Production from Alkali-Catalyzed Transesterification of Tamarindus indica Seed Oil and Optimization of Process Conditions. Molecules.

[36] Kelani R.O., Ahmad Z., Patle D. (2023). Mechanistic model-based control of biodiesel production processes: A review of needs and scopes. Chem. Eng. Commun..

[37] Karimi K., Saidi M., Moradi P., Najafabadi A.T. (2023). Biodiesel production from Nannochloropsis microalgal biomass-derived oil: Experimental and theoretical study using RSM-CCD approach. Can. J. Chem. Eng..

[38] Khalifa A., Faried M., Abdelsalam E.M., Samer M., Moselhy M.A., Elsayed H., Attia Y.A. (2024). Photoactivation of nano MgO anchored g-C_3_N_4_ enhances biodiesel production in Chlorella sorokiniana: A sustainable approach. Environ. Prog. Sustain. Energy.

[39] Mello B.S.D., Pozzi A., Rodrigues B.C.G., Costa M.A.M., Sarti A. (2024). Anaerobic digestion of crude glycerol from biodiesel production for biogas generation: Process optimization and pilot scale operation. Environ. Res..

[40] Wang F., Li Y., Wang Y., Xu X., Cao X., Cao T., Zhang W., Liu W. (2024). Engineering lipase TLL to improve its acid tolerance and its biosynthesis in Trichoderma reesei for biodiesel production from acidified oil. Bioresour. Technol..

[41] Siow H.S., Sudesh K., Ganesan S. (2024). Insect oil to fuel: Optimizing biodiesel production from mealworm (*Tenebrio molitor*) oil using response surface methodology. Fuel.

[42] Asif M., Javed F., Younas M., Gillani M.A., Zimmerman W.B., Rehman F. (2024). Investigating biodiesel production from chicken fat oil using bi-functional catalysts and microbubble mediated mass transfer. Fuel.

[43] Saidi M., Amirnia R. (2024). Recent advances on application of metal-organic framework-based catalysts in biodiesel production process: A review of catalyst types and activity, challenges and opportunities. Fuel.

[44] Atashkar H., Saidi M. (2024). Green biodiesel production from Nannochloropsis microalgae-derived oil using ZnAl-LDH catalyst: Process optimization and kinetic study. Fuel.

[45] Mansoorsamaei Z., Mowla D., Esmaeilzadeh F., Dashtian K. (2024). Sustainable biodiesel production from waste cooking oil using banana peel biochar-Fe_2_O_3_/Fe_2_K_6_O_5_ magnetic catalyst. Fuel.

[46] Annal U.N., Vaithiyanathan R., Natarajan A., Rajadurai V., Kumar P.S.M., Li Y.Y. (2024). Electrolytic biodiesel production from spent coffee grounds: Optimization through response surface methodology and artificial neural network. J. Taiwan Inst. Chem. Eng..

[47] Wajeh M.E., Granderath M., Mitsos A., Mhamdi A. (2024). Distributed economic nonlinear model predictive control for flexible electrified biodiesel production—Part II: Sequential and iterative architectures with computational delay compensation. Ind. Eng. Chem. Res..

[48] Betiku E., Oraegbunam J.C., Falowo O.A., Ojumu T.V., Latinwo L.M. (2024). Sustainable microwave-supported biodiesel production using sandbox oil and its waste shell as a nanoparticle green alkali heterogeneous catalyst. Process Biochem..

[49] Yang Z., Chen J., Tang B., Lu Y., Ho S.H., Wang Y., Chen C., Shen L. (2024). Metabolic interpretation of NaCl stress-induced lipid accumulation in microalgae for promising biodiesel production with saline wastewater. Chem. Eng. Sci..

[50] Chen Y., Yu H., Liu C. (2024). Xie, J.; Han, J.; Dai, H. Synergistic fusion of physical modeling and data-driven approaches for parameter inference to enzymatic biodiesel production system. Appl. Energy.

[51] Melchiorre M., Amoresano A., Budzelaar P.H.M., Cucciolito M.E., Mocerino F., Pinto G., Ruffo F., Tuzi A., Esposito R. (2022). Parts-Per-Million (Salen)Fe(III) Homogeneous Catalysts for the Production of Biodiesel from Waste Cooking Oils. Catal. Lett..

[52] Sun C., Hu Y., Sun F., Sun Y., Song G., Chang H., Lunprom S. (2022). Comparison of biodiesel production using a novel porous Zn/Al/Co complex oxide prepared from different methods: Physicochemical properties, reaction kinetic and thermodynamic studies. Renew. Energy.

[53] Huda M.S., Odegaard M., Chandra Sarker N., Webster D.C., Monono E. (2024). Enhancing Recovery Yield of Vegetable Oil Methyl Ester for Bioresin Production: A Comparison Study Using Acid Neutralization. ChemEngineering.

