# Supplementary material for: Comparison of High-Efficiency MgO/Na2CO3 and MgO/K2CO3 as Heterogeneous Solid Base Catalysts for Biodiesel Production from Soybean Oil
Source: Molecules. 2025 Jul 7;30(13):2876. doi: 10.3390/molecules30132876 (PMC12251140; doi:10.3390/molecules30132876)
Supplement: Supplementary file 1 [file molecules-30-02876-s001.zip › molecules-3705841-supplementary.pdf]

# Supplementary Materials

**(1) Determination of standard glycerol concentration–absorbance relationship**

In order to determine the content of glycerol in biodiesel samples, the relationship between standard glycerol concentration and absorbance was experimentally established. We accurately weighed 12.50g of pure glycerol reagent, which was dissolved in deionized water, transferred to a 250mL volumetric flask, and fixed with deionized water to configure 0.050g/mL of glycerol standard reagent. Then, 4.0mL, 6.0mL, 8.0mL, 10.0mL, 12.0mL, 14.0mL, 16.0mL, and 18.0mL of glycerol standard reagent were aspirated, transferred to a 100mL volumetric flask, and fixed with deionized water to obtain concentrations of 0.002g/mL, 0.003g/mL, 0.004g/mL, 0.005g/mL, and 0.006g/mL, 0.007g/mL, 0.008g/mL, and 0.009g/mL of glycerol standard solution, respectively. Meanwhile, 5wt% NaOH standard solution and 5wt% $CuSO_4{\cdot}5H_2O$ standard solution were prepared by dissolving solid sodium hydroxide (NaOH) and solid copper(II) sulfate pentahydrate ($CuSO_4{\cdot}5H_2O$), respectively, in deionized water. Sequentially, 1.0 mL of $CuSO_4$ standard solution and 3.5 mL of NaOH standard solution were pipetted into an empty conical flask, mixed, and shaken for 20 minutes. Then, 10 mL of 0.002 g/mL standard glycerol reagent was aspirated and added to the conical flask and shaken for 10 min to obtain a copper glycerol suspension. This step was repeated to prepare different concentrations of copper glycerol suspensions with different concentrations of standard glycerol solutions (0.002g/mL to 0.009g/mL), and 10mL of deionized water was used instead of the glycerol solution for the reference group. The obtained copper glycerol suspensions of different concentrations were filtered at atmospheric pressure (care was taken not to wet the filter paper with deionised water) to obtain copper glycerol standard solutions of different concentrations. The filtered solids are presumed to consist primarily of copper hydroxide precipitates, which exhibit slight solubility and are commonly formed under the alkaline conditions employed in the reaction. Considering the water solubility of glycerol components and the proposed reaction mechanism, it is reasonable to assume that these components remain entirely in the filtrate, with none retained in the solid phase. Although no further characterization has been performed to date, future studies utilizing techniques such as XRD or FTIR could be conducted

to confirm the precise composition of the precipitates.The maximum absorption peak of the copper-glycerol complex at approximate 625 nm was determined by full wavelength scanning. The scanning speed was 960 nm/min, and the spacing was 1.0 nm. The UV-Vis absorbance spectrum of copper-glycerol, obtained using a standard glycerol solution, is presented in Figure S1.

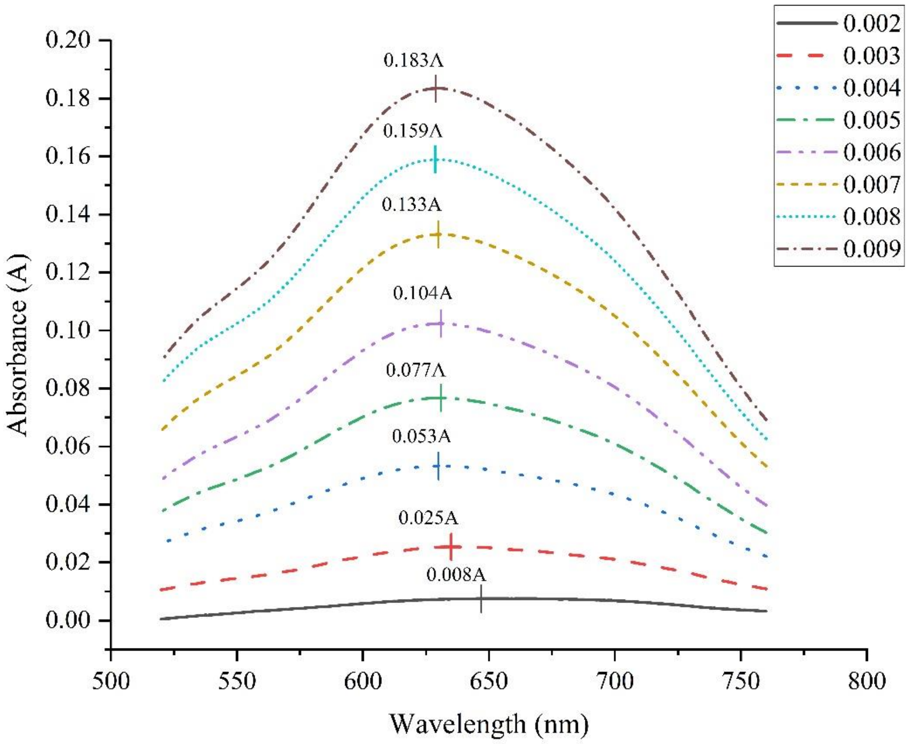


Figure S1: UV-Vis absorbance spectrum of standard copper-glycerol solution

By measuring the absorbance of glycerol copper solutions prepared from standard glycerol solutions at varying concentrations, a standard curve was constructed to represent the relationship between glycerol concentration and absorbance. After excluding the data point corresponding to the 0.002 g/mL (0.2 wt%) standard glycerol solution, which deviated from the expected trend, a strong linear correlation between glycerol concentration and absorbance was established, with an $R^2$ value exceeding 0.999, as illustrated in Figure S2.

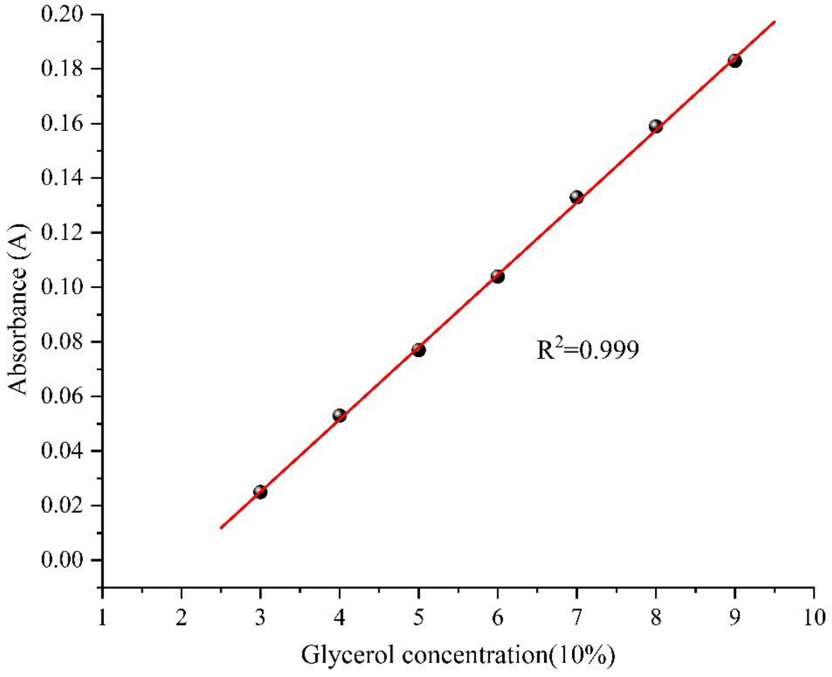


Figure S2. Absorbance-concentration calibration of standard copper-glycerol complex

This standard calibration curve can be effectively utilized for the quantitative analysis of glycerol content in subsequent biodiesel samples. To determine the parameter $\alpha$, which represents the purity coefficient of glycerol. The placement diagram of the glycerol standard reagent is shown in Figure S3.

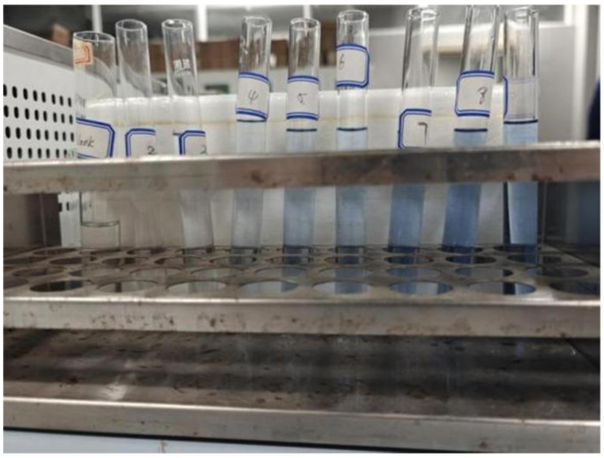

Figure S3. Glycerol-standard reagent placement.

**(2) Determination of FAME Yield**

Here, we assume that the main component of soybean oil involved in the reaction is triglyceride, and that biodiesel is primarily produced through the transesterification reaction between triglyceride and methanol.

The transesterification reaction proceeds according to the stoichiometric pathway illustrated in Scheme (1)

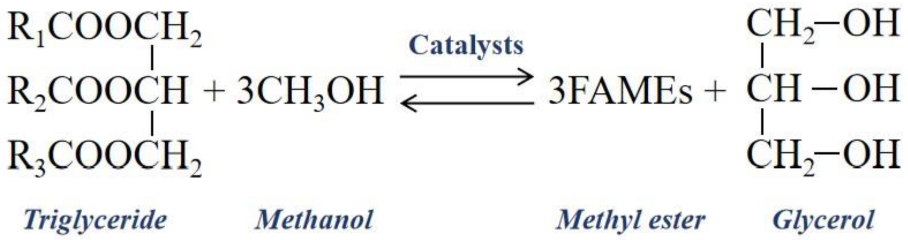


(1)

The biodiesel yield was calculated based on the glycerol byproduct quantified via a copper-glycerol complex spectrophotometric method. Glycerol reacts with $Cu^{2+}$ in an alkaline solution to form a blue complex exhibiting a maximum absorbance near 625 nm. The FAME yield formula is shown in Equation (2).

$$\text{FAME Yield}(\%) = \left(\frac{\alpha \times m_1/M_{glycerol}}{m_2/M_{soy}}\right) \times 100\% \qquad (2)$$

where $m_1$ and $m_2$ represent the mass of glycerol (g) and soybean oil (g), respectively. $M_{glycerol}$ and $M_{soy}$ represent the average molar masses of glycerol (92 g/mol) and soybean oil (880 g/mol), respectively. The molecular weight of soybean oil was estimated to be 880 g/mol, which falls within the commonly reported range of 872–885 g/mol for triglycerides derived from soybean oil. This value was selected as a representative average for calculation purposes, with the estimation error controlled within 1% [52,53]. The parameter $\alpha$ denotes the purity coefficient of glycerol, which reflects the actual concentration of glycerol in the crude glycerol product and is calculated using the method described previously.

**Select the result of a certain condition reaction as an example for our yield calculation:**

(Reaction condition: catalyst: 35-$Na_2CO_3$/MgO-600; temperature = 60 °C; catalyst consumption = 4.0 wt%; methanol-to-oil ratio = 18:1; reaction time = 2 h; mass of

Soybean oil: 16.0g)

After the transesterification reaction, crude glycerol was obtained through centrifugation, vacuum distillation, and liquid separation, with a final mass of 1.82 g. Take 1.0 g of crude glycerol sample and prepare a 1 wt% crude glycerol-water solution. Subsequently, the maximum absorption wavelength was determined to be 0.158 A by using the copper–glycerol colorimetric method. Based on the standard glycerol concentration-absorbance curve (Fig. S2), The glycerol content in the crude glycerol sample was determined to be 80.15%, which was used to obtain the parameter

$$\alpha = 80.15\%$$

Therefore, the FAME yield (%) can be calculated using Equation (2):

$$\text{FAME Yield}(\%) = \left(\frac{0.8015\times\frac{1.82}{92}}{\frac{16.0}{880}}\right)\times 100\%$$

$$\text{FAME Yield}(\%) = 87.21\%$$

**(3) Method Limitations**

Although the copper-glycerol colorimetric method offers a convenient means to quantify glycerol and infer biodiesel production, several important limitations should be noted:

**1. Incomplete monitoring of reaction intermediates.** A fundamental limitation of this method arises from the stepwise nature of the transesterification reaction: triglyceride → diglyceride (DG) → monoglyceride (MG) → glycerol, with each step producing one FAME molecule. [51] Notably, only the final step (MG → glycerol) generates free glycerol, which is the sole target of the current colorimetric assay. As a result, any FAMEs formed during the initial two steps remain unaccounted for. This means that, under conditions of incomplete conversion—where mono- and diglycerides may accumulate—the method may lead to inaccuracies in the calculated biodiesel yield. This selectivity for free glycerol, while convenient, represents an intrinsic limitation of the approach.

**2.Calibration range and reproducibility.** The standard curve excluded the lowest concentration point (0.002 g/mL) to achieve linearity ($R^2$>0.999), reducing sensitivity at

low glycerol levels and potentially biasing $\alpha$ (purity coefficient) determination. Furthermore, the formation of a copper–glycerol suspension suggests the presence of insoluble species. The filtered solids, which are presumed to be primarily copper hydroxide precipitates based on their characteristic appearance and known reaction conditions, are not expected to contain glycerol. Their removal by filtration therefore does not affect the accuracy of glycerol concentration measurements. Nonetheless, further characterization such as XRD or FTIR may be conducted in future work to confirm their precise composition.

Therefore, the copper–glycerol colorimetric method represents a rapid and cost-effective approach for assessing biodiesel yield. In this study, it was employed for the first time; however, the aforementioned methodological limitations may introduce certain inaccuracies into the yield measurements.Recommendations for future improvement work include supplementing glycerol-based estimates with direct FAME quantification (e.g., GC-FID) and monitoring intermediate mono-/diglycerides (e.g., HPLC) to obtain a comprehensive profile of reaction progress and validate glycerol-derived conversions.

**(4) Experimental apparatus**

The experimental instrumentation is shown in Table 1, and the key operating parameters are added as follows:
Centrifuge (TGL-16M): rotational speed, 8000 rpm; centrifugation time, 10 min.
Tube furnace (OTF-1200X-S): temperature control accuracy, ±2 °C; heating rate, 5 °C/min (Hefei Kejing Material Technology Co., Ltd.).
Mechanical stirrer: stirring speed set to 500 rpm (IKA RCT basic S000).
The main experimental instruments utilized in this study are summarized in Table S1.

Table S1. List of experimental instruments and key parameters.

| Instrument Name | Model | Manufacturer | Key Parameters |
|---|---|---|---|
| Reactor | 2000 mL | High Pressure Laboratory Equipment Factory | Pressure Resistance: 10 MPa, Material: 316L |
| UV–Visible Spectrophotometer | UV-1800 | Shanghai Yiheng Instrument Co., Ltd. | Wavelength Range: 190-1100 nm |
| Constant-Temperature Water Bath | DHG-9070A | Shanghai Box Instrument Co., Ltd. | Temperature Control Range: RT-300℃ |
| Electronic Balance | MX204 | Mettler Toledo, HongKong, China | Precision: 0.1 mg |
| pH Meter | PHS-3C | Beijing Jingke Instrument Co., Ltd. | Measurement Range: 0-14 pH |
| Centrifuge | TGL-16M | Shanghai Aopu Instrument Co., Ltd. | Max Speed: 16,000 rpm |
| Constant-Temperature Oven | DZF-6020 | Shanghai Qingpu Technology Co., Ltd. | Temperature Accuracy: ±1℃ |
| Ultrasonic Cleaner | KQ-250DE | Shenzhen Kaike Electronics Co., Ltd. | Power: 250 W, Frequency: 40 kHz |
| Scanning Electron Microscope (SEM) | SU8010 | Hitachi, Japan | Resolution: 1.0 nm @ 15 kV |
| Ion Sputter Coater | E-1045 | Hitachi, Japan | Gold Coating Thickness: 5 nm |
